# X-ray snapshots reveal conformational influence on active site ligation during metalloprotein folding†
†Electronic supplementary information (ESI) available: Details of (1) the experimental setup, (2) XTA data preprocessing, (3) XTA data analysis (SVD and GA), (4) XANES simulation, (5) estimation of excited state fraction and spectral reconstruction, (6) *k*-space EXAFS signal, (7) EXAFS analysis, (8) TRXSS solvent subtraction, (9) GA of TRXSS data, (10) Guinier analysis, and (11) BIFT analysis, along with 16 supplementary figures. See DOI: 10.1039/c9sc02630d


**DOI:** 10.1039/c9sc02630d

**Published:** 2019-09-03

**Authors:** Darren J. Hsu, Denis Leshchev, Dolev Rimmerman, Jiyun Hong, Matthew S. Kelley, Irina Kosheleva, Xiaoyi Zhang, Lin X. Chen

**Affiliations:** a Department of Chemistry , Northwestern University , Evanston , Illinois 60208 , USA . Email: l-chen@northwestern.edu; b Center for Advanced Radiation Sources , The University of Chicago , Illinois 60637 , USA; c X-ray Sciences Division of the Advanced Photon Source , Argonne National Laboratory , Argonne , Illinois 60439 , USA

## Abstract

Cytochrome *c* (cyt *c*) has long been utilized as a model system to study metalloprotein folding dynamics and the interplay between active site ligation and tertiary structure. However, recent reports regarding the weakness of the native Fe(ii)–S bond (Fe–Met80) call into question the role of the active site ligation in the protein folding process. In order to investigate the interplay between protein conformation and active site structures, we directly tracked the evolution of both during a photolysis-induced folding reaction using X-ray transient absorption spectroscopy and time-resolved X-ray solution scattering techniques. We observe an intermediate Fe–Met80 species appearing on ∼2 μs timescale, which should not be sustained without stabilization from the folded protein structure. We also observe the appearance of a new active site intermediate: a weakly interacting Fe–H_2_O state. As both intermediates require stabilization of weak metal–ligand interactions, we surmise the existence of a local structure within the unfolded protein that protects and limits the movement of the ligands, similar to the entatic state found in the native cyt *c* fold. Furthermore, we observe that in some of the unfolded ensemble, the local stabilizing structure is lost, leading to expansion of the unfolded protein structure and misligation to His26/His33 residues.

## Abstract 2

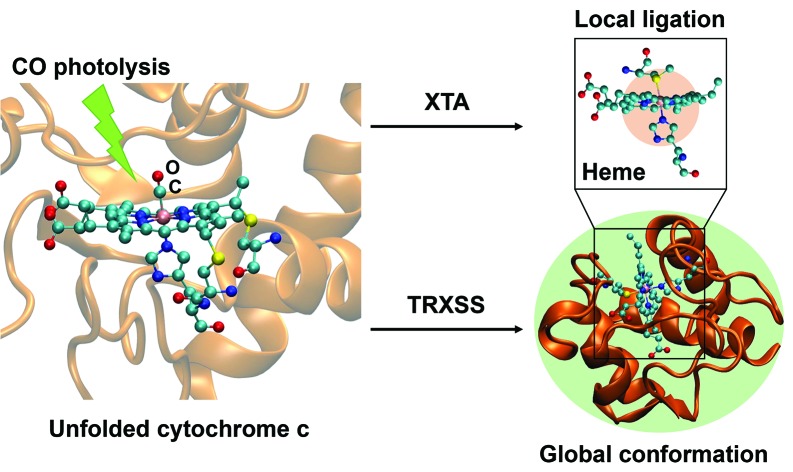
Parameters of local heme structure and overall conformation are tracked to reveal conformational influences on ligation states.

## Introduction

Protein functions are commonly established through their three-dimensional conformation, which is achieved through folding – a process that is driven by a delicate balance of forces arising from hydrogen bonding, electrostatics, hydrophobicity, and interactions with the environment.^1^ A perturbation to the balance between these forces may change the protein conformation and folding state, and in turn, regulate functions. In certain cases, a change of conformation can lead to misfolding, which often results in protein aggregation and precipitation, processes commonly involved in neurodegenerative disorders.^2^ While most protein structures are controlled by interactions between the constituent amino acids and their environments, metalloproteins have additional forces arising from the metal–ligand coordination, which anchor cofactors and amino acids to metal sites in the protein and therefore participate in determining the protein tertiary structure.^3^,^4^ As such ligation state changes are frequently involved in regulating native metalloprotein function, it is crucial to understand the interplay between transient backbone conformational dynamics and ligand binding properties.^5^

**Fig. 1:**
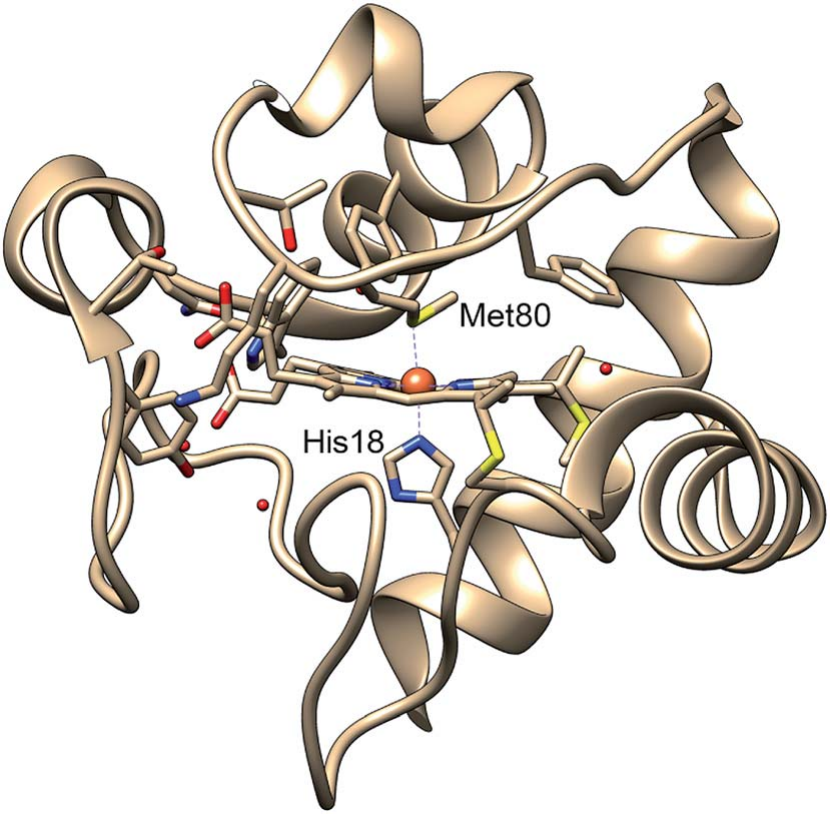
Structure of cytochrome *c* based on crystal structure (PDB entry ; 1HRC 9). In the native state, the Met80 and His18 residues are ligated to the heme iron, allowing the protein to act in the mitochondrial electron transport chain.

Cytochrome *c* (cyt *c*) is a 104-residue heme protein with dual functions in both mitochondria electron transport chain and cell apoptosis. The former function involves cyt *c* adopting a folded state with six atoms coordinating to the iron center forming an octahedral environment: four nitrogens from the protoporphyrin IX, a nitrogen from His18, and a sulfur from Met80 (Fig. 1). The latter function, related to apoptosis,^6^ involves the loss of the iron–sulfur bond, which allows cyt *c* to function as a peroxidase.^7^ The regulation of the dual functions of cyt *c* is carried out through the stability of the Fe–S bond, which has been observed to be sensitive to the protein tertiary structure. Specifically, the bond strength of Fe(ii)–S itself is on the order of thermal fluctuation (Δ*H* = 2.6 kcal mol^–1^) and should therefore not allow a stable ligation in the reduced native folded state.^5^ However, recent experiments on folded cyt *c* derived from ultrafast X-ray spectroscopy^5^ and computational simulations^8^ have indicated that the Fe(ii)–S bond is sustained by a ∼4 kcal mol^–1^ entatic stabilization provided by the protein structure, likely a hydrogen bond network. While the entatic state considerations have clarified how the Fe(ii)–S bond is maintained, it is still unknown what degree of tertiary structure folding is required to achieve the entatic stabilization, and whether partially unfolded states can maintain the native bond.

Cyt *c* has historically been utilized as a model system for investigation of the interplay between the active site and the protein conformation, as the strong interactions between the heme and the protein backbone regulate the protein structure and function.^4^,^10^ One of the most common methods to investigate the folding dynamics of cyt *c* is by photolysis of carbon monoxide (CO) ligated to the reduced heme of the protein. In CO-bound cyt *c* the native Met80 ligation is replaced by CO ligand under 4–5 M guanidine hydrochloride (GuHCl) denaturant condition, resulting in the protein assuming a partially unfolded state.^11^ By exciting the π–π* transition of the heme, CO dissociates from the heme in <1 ps,^12^,^13^ which triggers binding of other residues to the vacant site and associated conformational changes in the protein. Characterization of ligand binding and folding kinetics triggered by CO photolysis has been previously carried out with various indirect probing techniques such as optical transient absorption (OTA),^11^,^14^,^15^ time-resolved tryptophan fluorescence,^16^ time-resolved circular dichroism (TRCD),^17^,^18^ time-resolved magnetic circular dichroism (TRMCD),^18^ and transient grating (TG).^15^

The general folding pathway observed following CO photolysis is believed to involve some of the unfolded population adopting Fe(ii)–Met80 ligation on a timescale of 2–40 μs, while other portions of the unfolded population undergo Fe–His26/33 nonnative ligation (misligation) on timescales of 40–400 μs. The Fe(ii)–Met80 ligated population then proceeds to form the native state structure on a timescale of 200 ms to 1 s. However, despite the plethora of methods used to probe the kinetics of cyt *c* folding following photolysis, it is still unknown how the Fe(ii)–S bond forms so quickly in a purportedly unfolded structure. Furthermore, it is not well understood how the intermediate structures differ from each other in the tertiary fold to allow for the different ligations to occur.

In order to answer these questions, we utilized two parallel time-resolved techniques that can directly characterize both active site and tertiary folded structures. We perform X-ray transient absorption (XTA) spectroscopy and time-resolved X-ray solution scattering (TRXSS) to investigate the time evolution of the active site structure and the backbone structure, respectively, and discuss how the interplay between local and global structure affects the protein's folding process. XTA is a compelling method to directly observe local metal active site structure by specifically probing the metal center electronic state and the geometry of its surrounding atoms.^19^–^21^ This method has primarily been applied to study myoglobin and its model complexes,^22^,^23^ including those initiated by CO photolysis,^24^,^25^ in order to assign electronic transitions and determine bond distances between the heme iron and both the porphyrin nitrogens and axial ligands. Whereas these studies focused on local ligand dynamics, the current work expands the applications of XTA spectroscopy towards studies of protein folding, as well as new metalloproteins beyond myoglobin. TRXSS, on the other hand, offers a method to directly probe the overall folding state of the protein in solution. The method allows for direct investigation of low-resolution structure (in folded proteins),^26^ radius of gyration^27^ and flexibility of the protein.^28^ TRXSS is especially suitable for the current study because the signal arises directly from the changes in the protein structure. Moreover, it allows data acquisition in solution phase, which does not limit the conformational space of protein, unlike crystallographic methods. The findings in XTA and TRXSS experiments are linked to reveal how the native active site is stabilized, and the degree of protein unfolding that is required to maintain the stabilizing effect during folding.

## Experimental methods

### Sample preparation

Equine heart cyt *c* was purchased from Sigma-Aldrich and used without further purification. Cyt *c* was dissolved in a buffer with 50 mM phosphate and 4.0 M GuHCl at a concentration of 6 mg mL^–1^ (∼0.5 mM), with the exception that an 18 mg mL^–1^ concentration was used to probe time delays <4 μs in the TRXSS experiment. The pH value was adjusted to 7.0 using a small amount of 1 M hydrochloric acid or sodium hydroxide solution. A few drops of polypropylene glycol (PPG) were added to suppress foam formation. The solution was first purged with nitrogen for 25 minutes to remove oxygen. Sodium hydrosulfite was then added in excess to ensure complete reduction of cyt *c*. Finally, the solution was bubbled with pure carbon monoxide (CO) for 30 minutes before the experiments started to convert cyt *c* to CO-bound cyt *c*. At this temperature and GuHCl concentration, CO-bound cyt *c* is mostly unfolded, while the CO-free cyt *c* is fully folded.^29^

### X-ray transient absorption (XTA) measurements

XTA was performed at beamline 11-ID-D of Advanced Photon Source (APS), Argonne National Laboratory. A detailed instrument design has been described elsewhere.^24^,^25^ The sample was excited with a 527 nm laser pulse at the Q-band of the heme in cyt *c*.^11^ The 3 kHz, 527 nm laser pulse was generated the same way as reported in the previous publication done at the same beamline.^30^ The sample was probed by monochromatic X-ray pulses (6.536 MHz, ∼80 ps fwhm) in a standard 24-bunch operating mode with ∼10^6^ photons per pulse evenly separated by 153 ns. The excited volume exits the probing point as the sample jet flows. Throughout experiments, the solution was kept under CO environment by gently bubbling CO through the solution. The sample integrity was monitored by comparing the two pre-edge peaks of CO-bound cyt *c* to the reference scan, and samples were replaced promptly once a deviation with the reference occurs. We separately measured the steady-state XANES spectrum for the native conformation and ligation states with Fe(ii) heme, which matches well with the spectrum reported in literature.^31^

The difference signal (laser-on–laser-off) was decomposed using Singular Value Decomposition (SVD) method, which left spectral components that have kinetic traces with mixed time constants. We further utilized Global Analysis (GA) which requires the spectral components to evolve following a kinetic model. The resulting species-associated difference spectra were then assigned as in the Results section. During assignment, we simulated the difference signal using FEFF9.6 (ref. 32) software and compared it to the species-associated difference spectra. Details of the experiment, data processing, GA, and simulation can be found in ESI.†

### Time-resolved X-ray solution scattering (TRXSS) measurements

TRXSS was performed at BioCARS 14-ID-B beamline of APS. The pump–probe TRXSS experiment setup and data acquisition methodology at BioCARS have been published previously.^33^–^35^ To reduce oxidation, the sample was kept in a reservoir with a nitrogen flow over the surface. The sample was delivered by a syringe pump into a custom built, temperature-controlled capillary flow cell.^34^ The Q-band was excited by laser pulses with a pulse duration of 7 ns at 532 nm.^11^ The measurements were performed at 25 °C. The full detail of the experiment, including data analysis methods, can be found in ESI.†

## Results

### Overview of raw data

**Fig. 2:**
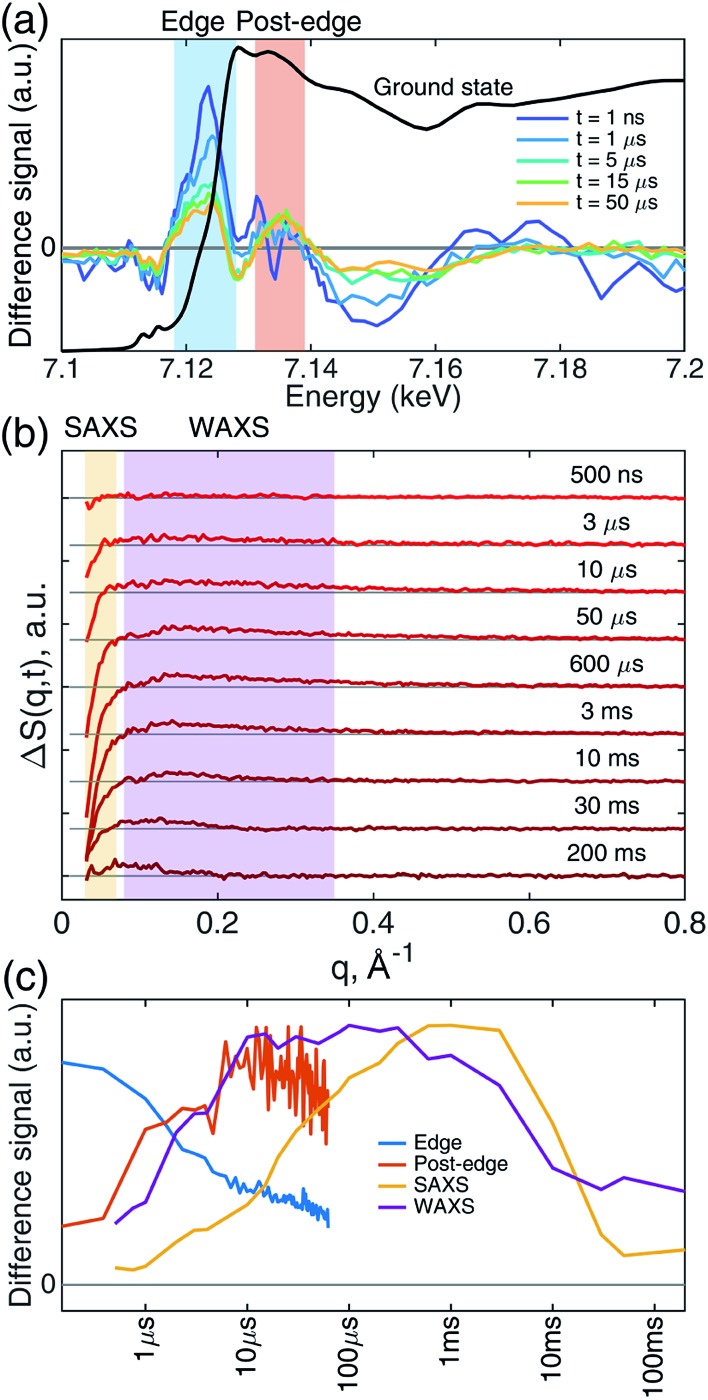
XTA and TRXSS difference signals show complex evolution. (a) XTA difference signal at representative time delays in the iron K-edge XANES region. Difference signal was calculated by subtracting the ground state laser-off signal from the laser-on signal (laser-on–laser-off). The 1 ns signal is smoothed with three-point moving averaging along the energy axis; for other time delays, signals represent the average of individual spectra recorded within ∼1 μs time window centered at the time delays shown in the legend. The ground (Fe–CO) state (black) XANES spectrum is included as a reference. Kinetic traces of the integrated signal intensities in the blue (edge region, 7118–7128 eV) and red (post-edge region, 7131–7139 eV) shaded area are drawn in (c) with corresponding colors. (b) Buffer-free TRXSS difference signal at representative time delays. Regions highlighted with yellow and purple shades represent SAXS (0.03 < *q* < 0.07 Å^–1^) and WAXS (0.08 < *q* < 0.35 Å^–1^) regions whose integrated intensity are shown in (c). (c) Integrated XTA and TRXSS difference intensities as a function of time. Points on the XTA traces are average values of 6 bunches except for those <1 μs, where 3 bunches are averaged. The color code of the traces corresponds to the regions of interest indicated in panels (a) and (b).

Transient iron K-edge X-ray absorption signals as a function of the delay time between the laser excitation and X-ray probe pulses from 1 ns and 60 μs were collected with a sampling period of 153 ns defined by the X-ray pulse train from APS at the repetition rate of 6.536 MHz. The time evolution of the resulting difference XANES signals (laser-on–laser-off) at select time delays, along with the ground (Fe–CO) state, are shown in Fig. 2a, showing the most pronounced difference signals at certain energy regions where the reaction kinetics were extracted and analyzed. The XTA difference signal at 7124 eV corresponds to the red-shift of the iron K-edge from that of the ground state spectrum. The XTA difference signal at 7135 eV, reflects the intensity change of the second peak after the edge. The kinetics traces extracted from regions near these two energies are shown in Fig. 2c. Immediately after the excitation, the difference signal points to a large edge shift at 7124 eV. This peak sharply declines until about 10 μs, where it starts to decay slowly. In contrast, the 7135 eV trace shows growth until 10 μs, followed by a slower decay. While the signal evolution during the early sub-20 μs reflects the events corresponding to the ligation dynamics of the heme, the slower decay at later time delays corresponds to the excited sample flowing out from the probed region (see Experimental methods).

While the Fe(ii) heme active site structures were followed by XTA, the global protein conformation along the course of the refolding after the CO dissociation was followed by TRXSS. The scattering difference signals free of the buffer heating contribution were obtained by using standard procedures as in previous works (see ESI for details†).^34^–^37^ The resulting difference signals containing protein only contributions at selected time delays are shown in Fig. 2b. The kinetic traces obtained by the time evolution of the scattering signals integrated in the SAXS (0.03 < *q* < 0.07 Å^–1^) and the WAXS (0.08 < *q* < 0.35 Å^–1^) regions, respectively, revealed processes taking place on multiple time scales and different kinetics in the SAXS and WAXS regions (Fig. 2c). The WAXS signal rises from 500 ns up to 10 μs with a trajectory similar to that of 7.135 keV from XTA data and remains constant until 1 ms, which is followed by a decay up to 10 ms and plateauing on the 100 ms time scale. By contrast, the SAXS region indicates a loss of intensity appearing as a stepwise process in the time window between 10 μs and 1 ms, which is followed by a decay up to 50 ms and plateauing at later time scales.

**Scheme 1:**
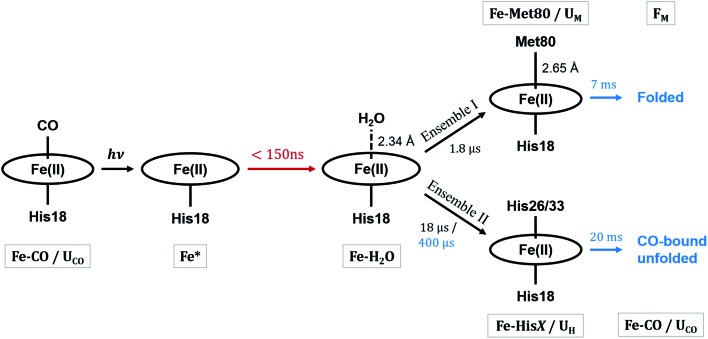
The kinetic model of cyt *c* folding. The kinetic and structural parameters are determined in this work. State names referred to in the text are below or above the structures with gray bounding boxes. The process marked in red is observed only in XTA, while processes in blue are observed only in TRXSS, due to timescale limitations of the instruments. Processes marked in black are observed utilizing both techniques. Ensembles are referred to in the Discussion.

These observations suggest that there are multiple local and global conformational states involved in the folding process. Previous spectroscopic works identified that Met80 binding happens on the time scale of <10 μs, histidine binding in between 10 μs and 1 ms, and overall folding about 10 ms.^11^,^15^,^17^,^18^,^35^,^38^,^39^ Since our raw data shows events with time scales in agreement with the literature values, we utilized a kinetic model derived from these assignments for our global analysis (GA), which is shown in Scheme 1. The model contains the initial photoproduct which undergoes two parallel paths leading to a native, methionine-bound state (Fe–Met80) and a non-native, histidine-bound (Fe–His*X*, *X* = 26 or 33) state, respectively. The Fe–Met80 state then forms the native folded conformation, while the Fe–His*X* simply decays back to the Fe–CO ground state. Compared to previous studies, we had to add an additional intermediate state, a cyt *c* with a penta-coordinated heme Fe*, which was observed based on the XTA results, as discussed below. The presented kinetic model was used in GA, allowing us to extract of time scales for each transition, as well as time-independent species-associated signals which are then used for modeling and structural assessment.

Since the two techniques probed structural dynamics on different time scales, GA were carried out separately. Based on the autocorrelation function for the left singular vector in singular value decomposition (SVD), which indicates two and three significant components for XTA and TRXSS datasets, respectively, we identified two species-associated absorption spectra and three species-associated difference scattering patterns after GA fitting (see ESI†). Additionally, the best fit values for the time scale of each transition obtained from GA are summarized in Scheme 1. Below we describe each component and assign their ligation and conformational states.

### X-ray transient absorption

#### The starting Fe–CO (ground) state

**Fig. 3:**
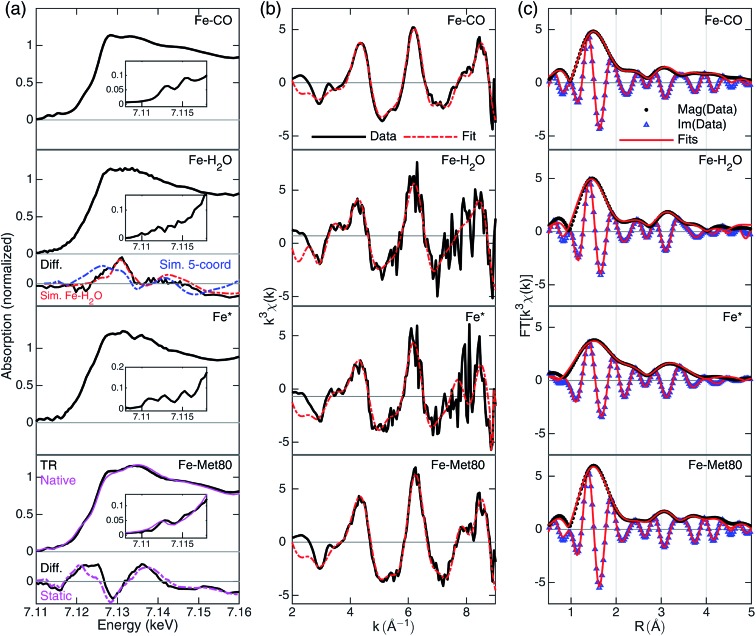
A summary of XTA results following photolysis of the CO-bound cyt *c*. Rows from top to bottom are data for Fe–CO, Fe–H_2_O, Fe* and Fe–Met80 states. (a) Reconstructed XANES spectra with the pre-edge features in the insets and the XANES difference signals derived from GA (black, “Diff.”) in the lower panels. For Fe–H_2_O state, the simulated differences for penta-coordinated (blue) and Fe–H_2_O (red) structures are included. For Fe–Met80 state, static measurements of reduced folded Fe(ii)–cyt *c*, (purple, “Native”) and the static difference (reduced folded Fe(ii)–cyt *c*–Fe–CO) are included (purple, “Static”). For details of the simulation and spectral reconstruction, see ESI.† (b) EXAFS spectra in *k*-space (black) and the fits (red). The *k*-space fitting windows are Fe–CO: 2.55–8.7 Å^–1^, Fe–H_2_O: 2.6–8.8 Å^–1^, Fe*: 2.6–7.36 Å^–1^, Fe–Met80: 2.7–8.9 Å^–1^. (c) Fourier-transformed EXAFS spectra in *R*-space with magnitude (black dots, “Mag”) and imaginary (blue triangles, “Im”) part along the fits to the data (red lines). *R*-Space fitting windows are 1–4.55 Å.

The Fe–CO state represents the starting ground state of the system, prior to the photolysis of the CO ligand. The XANES spectrum, *k*-space EXAFS spectrum, and *R*-space EXAFS spectrum of the Fe–CO state are shown in Fig. 3, top row. The XANES spectrum contains two pre-edge peaks at 7112.6 and 7115.1 eV, originating from the low spin Fe(ii) 3d^6^ configuration imposed by the strong field CO ligand. The peak positions and the energy splitting between the two peaks are common among Fe(ii)–CO heme compounds^25^,^40^ and proteins,^24^,^41^ with the lower energy peak assigned to the 1s → e_g_ transition and the other the 1s → π* transition.^40^,^42^,^43^ The transition edge energy, defined as the first inflection point of the spectrum, is at 7120.3 eV. The overall XANES spectral features and observed energies are in accordance with those observed for CO-bound myoglobin with a similar coordinating environment.^24^

**Scheme 2:**
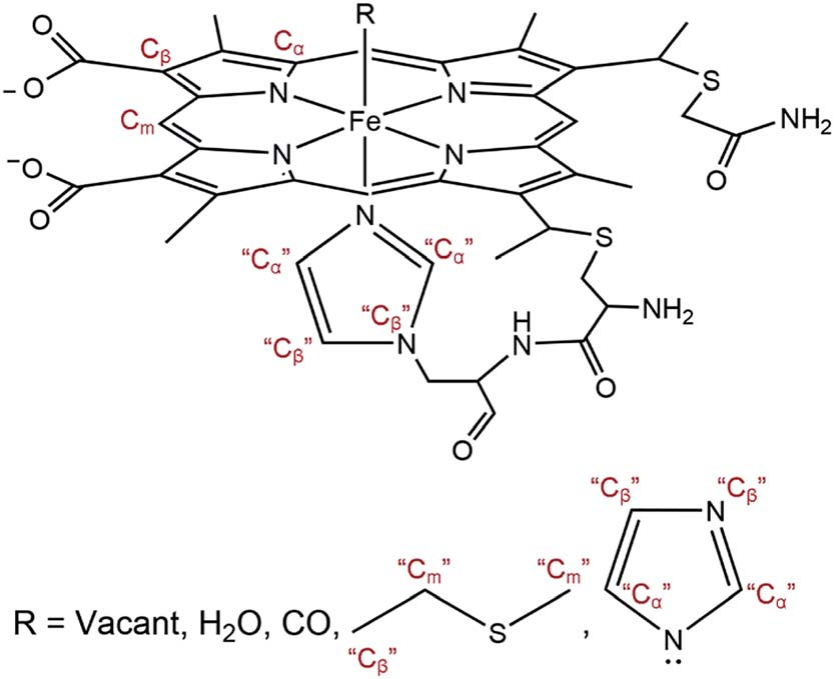
General model showing scattering paths included in EXAFS fitting. Based on the distance to the heme iron, atoms of the second shell are grouped into C_α_, C_m_, and C_β_ (see ESI†).

The EXAFS portions of the data were fitted with appropriate theoretical signals generated using FEFF in the ATHENA/ARTEMIS platform,^44^ using the heme structure from the Protein Data Bank entry ; 1HRC 9 as template (Scheme 2) to extract structural information. The details of the structural analysis are outlined in the ESI.† EXAFS fitting retrieved structural parameters (Table 1) that generally agree with those observed previously, with a 1.73 Å Fe–C(CO) distance.^45^

**Table 1:** EXAFS fitting parameters for each observed species. *S*_0_^2^ = 0.95, Δ*E* = –0.751 eV. For an estimation of bond length precision, see ESI

Fe–CO (CO-bound) (ground state)				Fe* (penta-coordinated) (*t* = 1 ns)				Fe–H_2_O (water-bound) (GA species 1)				Fe–Met80 (GA species 2)			
Path	CN	*R* (Å)	*σ* ^2^ (Å^2^)	Path	CN	*R* (Å)	*σ* ^2^ (Å^2^)	Path	CN	*R* (Å)	*σ* ^2^ (Å^2^)	Path	CN	*R* (Å)	*σ* ^2^ (Å^2^)
Fe–C	1	1.73	0.002												
Fe–O	1	2.89	0.001					Fe–O	1	2.34	0.005	Fe–S	1	2.65	0.008
Fe–N	5	2.02	0.005	Fe–N	5	2.05	0.007	Fe–N	5	2.05	0.005	Fe–N	5	2.03	0.005
Fe–C_α_	10	3.11	0.007	Fe–C_α_	10	3.06	0.009	Fe–C_α_	10	3.09	0.008	Fe–C_α_	10	3.08	0.007
Fe–C_m_	4	3.40	0.004	Fe–C_m_	4	3.43	0.002	Fe–C_m_	4	3.42	0.001	Fe–C_m_	6	3.37	0.006
Fe–C_β_	10	4.46	0.003	Fe–C_β_	10	4.48	0.001	Fe–C_β_	10	4.45	0.005	Fe–C_β_	11	4.46	0.002

#### The first GA species

The first species-associated signal resulted from GA has the highest population at the earliest time delays and monotonically decays with two time constants (2.1 ± 0.24 μs and 15 ± 8.0 μs). Since the previous studies reported no protein residues bind extensively to the heme at this short time delay, the possible heme structure for this species would be penta-coordinated or with a water axial ligation. We compared this signal to the FEFF-simulated XANES difference signals of the two candidate ligation states respect to the CO-ground state; the Fe–H_2_O signal matches better with the experimental difference spectrum than that from a penta-coordinated structure (Fig. 3a, second row, lower panel and Fig. S5; see ESI for simulation details†).

The reconstructed XANES spectrum of this state is shown in Fig. 3a, second row. The ground state (CO-bound) signal and the species-associated difference signal (Fig. 3a, lower panels) from GA with an excited state fraction of ∼0.49 (see ESI†) were used to reconstruct the total X-ray absorption spectrum. The XANES spectrum shows no apparent peaks in the pre-edge region which is in agreement with a more centrosymmetric Fe(ii) coordinating geometry. This geometry stands in contrast to the plausible penta-coordinated state, which contains centro-asymmetry due to the vacancy left by CO departure. The edge energy is at 7120.3 eV. It does not have an apparent shoulder at the edge (∼7123 eV), which again points to an octahedral geometry.^46^ The overall shape features enhanced intensity at 7128 eV, an indication that the Fe(ii) is still in a high-spin state.^47^ These observations suggest that the spectrum should be assigned to a Fe–H_2_O state.

Fittings of the *k*-space and *R*-space EXAFS spectra are shown in the third row of Fig. 3b and c, respectively. In EXAFS analysis, the oxygen was best fit to 2.34 Å from the heme iron (Table 1), indicating a weakly interacting ligation. The Fe–N distance is prolonged (2.05 Å) compared to the CO-bound state, signaling the well-known heme doming effect is present,^24^,^25^ and that the iron stays in high-spin state, consistent with the observations from XANES analysis. The second shell Fe–C_α_, Fe–C_m_, and Fe–C_β_ distances are close to those of Fe–CO state, indicating a rather rigid heme structure.

#### The Fe* state right after the CO dissociation

As the first GA species was assigned to a water-bound state, whether the water can enter the heme pocket and bind to heme at 1 ns (the first time point) becomes the next question. In Jones and coworkers' report, they argued there is no ligand binding in less than 10 ns.^11^ In similar CO photolysis studies done on myoglobin and hemoglobin chains, the entry rate of water to the distal side of the heme pocket was found to be on the order of 100 ns,^48^ two orders of magnitude slower than the earliest time delay in the XTA dataset. Therefore, we assumed that at 1 ns the heme is in a penta-coordinated (Fe*) state and separated the total signal using the excited state fraction for analysis (see ESI†).

The most apparent change in the XANES spectrum (Fig. 3a, third row) is the collapse of the two sharp pre-edge features and a significant red-shift of the Fe K-edge energy from those seen in the starting Fe–CO state. Several structural changes could contribute to the change, (1) the low-spin to high-spin transformation of Fe(ii) due to the departure of the strong field ligand CO, (2) the breakage of Fe–C(CO) bond, and (3) the disappearing π-backbonding from Fe(ii) to CO.^22^,^24^,^25^ The reconstructed XANES spectrum showed an edge estimated at 7117.9 eV, 2.4 eV lower than that in CO-bound and in Fe–H_2_O state. The shift in edge energy is expected when the electronic environment near iron switches from a low-spin, octahedral geometry to a high-spin, square pyramidal geometry.^25^,^46^,^47^ In addition, the shoulder at 7123 eV is also consistent with the signal from a square pyramidal coordination geometry.^46^

Fittings of the *k*-space and *R*-space EXAFS spectra are shown in the third row of Fig. 3b and c, respectively, and the structural parameters are summarized in Table 1. The Fe* state has elongated average Fe–N distances (from 2.02 Å to 2.05 Å), as expected from the heme doming observed in CO photolysis experiments on myoglobin.^24^,^25^ Longer Fe–N distances also suggest that the iron is in the high-spin state with electron occupation in the molecular orbitals with higher energies. Fe–C_α_ distance shortened from 3.11 Å to 3.06 Å, while Fe–C_m_ and Fe–C_β_ distances stayed largely unchanged, indicating some distortion of the macrocycle before returning to the values found in the Fe–H_2_O state.

#### The second GA species

The second species-associated XANES difference spectrum derived from GA (Fig. 3a, bottom row, lower panel) was assigned to a Fe–Met80 (methionine-bound) state due to the observation that this spectrum rises with a time constant of 2.1 ± 0.24 μs (Fig. S5 and ESI†) which agrees with previous optical spectroscopy results. The GA signal also matches the difference spectrum generated from static measurements of Fe–CO and folded Fe(ii)–cyt *c* (Fig. 3a, bottom row, purple lines). However, the XANES spectrum of this Fe–Met80 state differs from the native Fe(ii)–cyt *c* spectrum. The pre-edge feature is at 7112.8 eV, and the edge energy is at 7120.4 eV, both 0.6 eV higher than that in the folded state with the same set of coordinating atoms. The spectral shape resembles the native low-spin Fe(ii) state spectrum (Fig. 3a, bottom row, purple line) but with a distinct difference at 7128 eV in intensity. These findings indicate that the active site structure is different from that of the native state. Indeed, EXAFS analysis (bottom row of Fig. 3b and c and Table 1) revealed that in Fe–Met80 state, the Fe–S bond was best fit to an unusually long distance of 2.65 Å, 0.36 Å longer than in its native ligation.^31^ While the Fe–S distance can be >3 Å in a highly transient state on a picosecond timescale,^5^ most experimentally determined values for a heme-based iron–sulfur bond distance are within 2.2–2.5 Å.^31^,^45^,^49^–^51^ The Fe–N distance shortened from 2.05 Å to 2.03 Å, indicating the heme doming effect is less pronounced, which is in agreement with an octahedral geometry. The Fe–C_α_, Fe–C_m_, and Fe–C_β_ distances stayed roughly the same as those of CO-bound state (Table 1).

#### The Fe–His*X* state (see ESI†)

As previously mentioned, one of the pathways of cyt *c* following the CO photolysis involves the adoption of a His26/His33 ligated state in parallel to the evolution of a Met80 ligated state. However, the simulated XANES difference spectra for the Fe–H_2_O and Fe–His*X* states were found to be similar, which precluded the separation of a species-associated difference spectrum and subsequent structural analysis. Nonetheless, from the kinetic modeling, we retrieved a formation time constant of 15 ± 8 μs, which is in line with the value derived from TRXSS.

### Time resolved X-ray solution scattering (TRXSS)

**Fig. 4:**
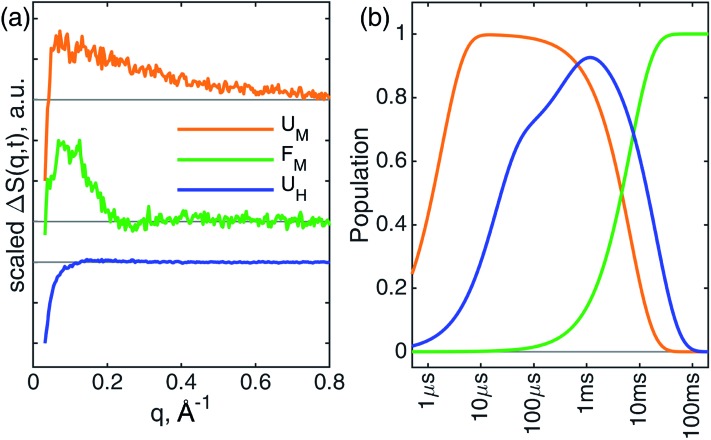
Global analysis result for TRXSS. (a) Species-associated kinetics derived from global analysis. (b) Species-associated difference scattering curves derived from global analysis. The curves are scaled for clarity.

The kinetics derived from global analysis suggest that an intermediate state is populated on a time scale of 1.8 ± 0.1 μs (Fig. 4, orange), which matches the XTA result for Met80 binding time scale. For this reason, we assign this early intermediate, U_M_, to a Met80-bound state. Qualitatively, the scattering difference curve for this intermediate species appears as a uniform increase of intensity in the WAXS region, which indicates secondary structure formation and partial folding. Guinier analysis of the difference signal^23^ indicates that U_M_ species are more compact with a radius of gyration of *R*_g_ = 18.2 ± 1.2 Å, compared to the ground CO-bound (U_CO_) state with *R*_g_ = 24.6 ± 0.4 Å (see ESI for details†). The partial folding of the protein is further corroborated by inspection of difference pair distribution function calculated using Bayesian Inverse Fourier Transform (BIFT),^52^–^54^ which exhibits a gain of electron density at <10 Å, indicating formation of secondary structure, and corresponding loss of electron density at longer distances, indicating the collapse of the protein (see ESI†). The observation of the collapsed state with TRXSS allows us to rule out burst phase folding into the native state.^55^

**Fig. 5:**
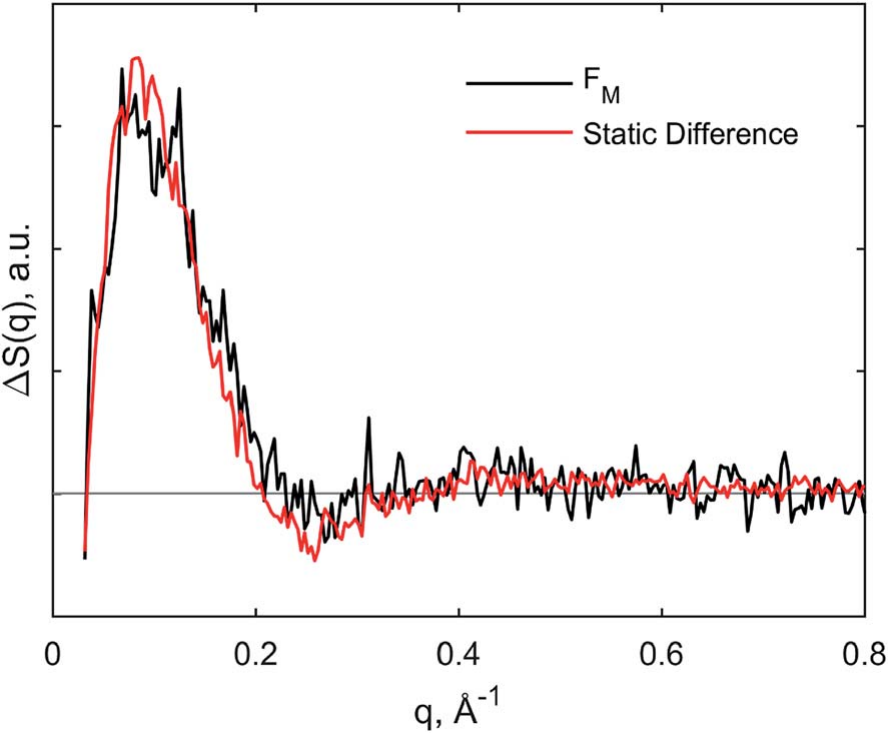
Comparison of species-associated scattering difference curve for F_M_ state with calculated difference signal obtained from static data.

The U_M_ state converts into another state with a time constant of 6.6 ± 0.7 ms (Fig. 4, green) as predicted by our kinetic model. The time constant is in agreement with the previous folding time scales derived from transient grating measurements.^15^ We compared the species-associated difference signal for this state with the static (steady-state) difference between folded cyt *c* and unfolded CO-bound cyt *c* (Fig. 5). Excellent agreement between the curves clearly confirms the correct assignment of the folded, F_M_, state. Interpretation of the F_M_ signal as folding is further corroborated by the analysis of the pair distribution function (see ESI†), which indicates a significant gain in the electron density at distances <30 Å and loss at larger distances. The *R*_g_ = 13.6 ± 0.3 Å extracted from the Guinier analysis for F_M_ state agrees with the values published in literature,^56^ and shows the extent of compaction of the folded state compared to the intermediate U_M_ state.

In parallel with the formation of U_M_, we observe the formation of U_H_ species-associated with His*X* (*X* = 26 or 33) binding to the heme (Fig. 4, blue). The best fit kinetic model has shown that the His*X* binding is a biphasic process with two time constants: 18 ± 1 μs and 400 ± 40 μs. Such complex His*X* binding kinetics have been reported previously in spectroscopic studies with similar time scales.^57^ Additionally, the early time scale for His*X* binding matches well with the XTA results discussed above. The biphasic binding of His*X* has been proposed to arise due to separate binding of His26 and His33, each of which taking different amounts of time for ligation. From the kinetic fitting we derive that the branching between fast and slow components is 0.66 ± 0.01. The U_H_ species decays back to the ground state with a single time constant of 20.4 ± 0.7 ms due to the heme rebinding with CO. The species-associated scattering patterns indicate a large decrease in SAXS intensity, which indicates that this pathway involves the protein adopting an expanded and more disordered state.^34^,^58^ Further unfolding of U_H_ species compared to U_CO_ is in line with the expanded denatured state assumed by oxidized cyt *c* with His*X* ligation in mild denaturing conditions, which has a *R*_g_ of ∼30 Å.^37^,^59^ The assignment of the U_H_ signal to unfolding is further corroborated by BIFT analysis, which indicates the loss of electron density at distances spanning up to maximum dimension *D*_max_ of U_CO_ (see ESI for details†).

### Overall folding scheme

Combining all information gathered above, the overall folding scheme, along with structural details near the heme, of cyt *c* after CO photolysis is summarized in Scheme 1 with the rate constants now determined from our data. After the photolysis, the transiently populated heme site likely assumes a domed structure, followed by weak coordination of a water molecule. From here, one of the split paths leads to the longer-than-native coordination from Met80, accompanied by a partial folding, which finally arrives at the native folded conformation. By contrast, on the other path a histidine binds to the heme while the protein expands further and never reaches the folded structure. The striking difference of the outcomes points to the subtle balance between the ligation and conformations, which will be discussed below.

## Discussion

### Interplay between conformational and ligation changes

Following the binding by water at the axial ligand site, some of the photolyzed protein population quickly adopts a Fe–Met80 ligation which also forms a partially folded structure with some secondary structure formation. Since both global analyses on XTA and TRXSS data yielded ∼2 μs time constants, we can link the concerted dynamics on the two spatial scales to assign the secondary structure formation as the backbone movement that pulls Met80 towards the heme. It is notable that the 2 μs Fe–S bond formation time constant here^11^,^15^,^18^,^39^ contrasts with the tens of milliseconds Fe–S formation times observed in the cyt *c* refolding induced by the reduction of Fe(iii) *via* an electron transfer process, where the starting structure is considerably more disordered.^60^ It is also faster than the expected diffusion timescale (about 35 μs) required for the formation of a loop between His18 and Met80 when the diffusion process is modeled as a peptide chain undergoing random walk.^38^,^61^ The drastic difference in the observed time constants suggests that Met80 is in proximity to the heme in the CO-bound ground state. However, the Fe–S distance in the Fe–Met80 state was determined by EXAFS to be 2.65 Å, much longer than 2.29 Å in the native conformation^31^ indicating a very weak interaction.^5^ Therefore, this state likely originates from a local structure that positions Met80 close to the heme, rather than a stable ligation from Met80 to the heme. Considering that this state ultimately forms the folded state, the local structure that supports the Met80 should be similar to that in the native conformation, namely the residues 65–85 that covers the distal side of the heme.

From the tertiary structure perspective, the Fe–Met80 state is partially folded, and the native state is only adopted on a much slower, millisecond timescale (6.6 ms^–1^). This disconnection between the formation rate of native active site ligation and native conformation therefore suggests further protein reorganization is required to fully form a stable Fe–S bond as in the native conformation, which has been suggested to be enabled by a hydrogen bond network from Tyr67 and nearby residues.^5^ TRCD studies suggest that the secondary structure other than the terminal helices, including 60's helix on which Tyr67 sits, does not form until at least 5 ms after photolysis.^17^ Our results also suggest that the structure that fully supports the native bond between Fe–S is established only during the later phase of the folding process and is in line with the recent description of protein stabilization by the hydrogen bond network.

### Heterogeneous ground state structures could lead to different ligations

The thermodynamic description at the heme active site in cyt *c* has recently come under scrutiny. While the Fe–S bond has been thought to be stable due to its presence in crystal structure, it was recently reported to be fairly weak (Δ*H* = 2.6 kcal mol^–1^).^8^ In contrast, the competing non-native ligands, His26 and His33, are not only more energetically favored (Δ*H* = 7.2 kcal mol^–1^)^8^ as pointed out by Density Functional Theory calculations, but closer in sequence to the other axial ligand to the heme, His18, making the diffusion-induced contact rate higher. Therefore, to form the native Fe–S bond, the protein must presumably prevent other ligands from approaching and displacing Met80.

Since the CO-bound cyt *c* is in the disordered state, many subpopulations exhibiting different energetic and kinetic profiles can exist simultaneously. As suggested by Latypov and coworkers, CO-bound cyt *c* could assume multiple disordered conformations.^62^ Such an ensemble of disordered states may explain the parallel folding processes observed in many of the previous time-resolved studies on CO-bound cyt *c*.^11^,^15^,^57^ After photolysis, the subpopulations with an intact local structure assume the Fe–Met80 ligation state, and the ligation is protected throughout the folding process. On the other hand, the subpopulations without an intact local structure should adopt a ligation state with lowest energy solely due to metal–ligand bond strength, in which case the Fe–His*X* ligations dominate.^8^,^63^ Consistent with our interpretation of Fe–His*X* state detected in TRXSS experiments, a release of the sequence consisting of residues from His26/33 to Glu104 at the C-terminal would generate a large expanding and unfolding signal, sampling a larger conformational space and gaining entropy. The Fe–His*X* ligation state is referred to as the misfolded “kinetic trap” during the folding process.^14^,^18^,^39^ We corroborate this notation by observing this ligation state being both enthalpically and entropically favored.

### Protein support for the Fe–H_2_O intermediate ligation state

A water-bound heme structure after photolysis, determined by XTA, seems to contradict the previous works suggesting that Fe(ii) does not bind water. In steady-state resonance Raman spectroscopy measurements, the non-native Fe(ii) heme coordination states detected are assigned to either a bis-His or a penta-coordinated state, but not a water-bound state.^64^,^65^ Even in CO photolysis studies, Jones and coworkers also attributed the immediate product after photolysis to a penta-coordinated species.^11^ However, it is possible that a water molecule stays bound to the heme transiently. In the TRCD study of CO-bound cyt *c*, Chen and coworkers could not completely rule out the water as a ligand for early ligation events.^17^ In ET-initiated cyt *c* folding experiments, the water was observed to dissociate from the Fe(ii) with a time constant of about 1 ms after photoreduction.^60^ Furthermore, the energy *required* for maintaining Fe(ii) and H_2_O in proximity was calculated to be 2.0 kcal mol^–1^,^8^ a relatively small amount. Finally, Esquerra and coworkers determined the spectral change of a water molecule entering the myoglobin and hemoglobin heme pockets after CO photolysis.^48^ The intensity of the difference spectrum is 25-fold smaller than that caused by the photolysis itself. Therefore, given the multiple events during folding of cyt *c* that could affect the optical absorption spectrum, the Fe* and Fe–H_2_O states discovered in this work may be indistinguishable in optical studies.

For this XTA-detected ligation state, there is little TRXSS structural difference (at 500 ns), which allows us to deduce that in the CO-bound state a portion of the residue structure around the heme is supportive of a Fe–O bond. In addition, the defined bond distance without a large Debye–Waller factor *σ*^2^ implies that it is unlikely that many water molecules exchange at the binding site. Questions then arise regarding how this water was held fixed in the proximity of the heme without a favorable ligation. The most probable scenario seems to be that the same local structure supporting Met80 is also responsible for the water molecule. In the crystal structure, the heme crevice of cyt *c* is arranged tightly with only one bound water allowed inside.^9^ Given some secondary structures remain in CO-bound cyt *c* under 4.6 M GuHCl, the heme pocket may not have unfolded completely. The spatial restriction may have forced a single water molecule to stay close to the heme after CO photolysis. An alternative explanation may be that the water molecule is stabilized by electrostatic effect. However, the heme does not attract water, and in the native state there is no charged group near the heme which is buried in the hydrophobic core, which renders this scenario unlikely. Yet another possibility is that the water molecule is stabilized by a hydrogen bond by forming a network as in the native state, but the associated energy for this interaction (a few kcal mol^–1^) is easily overwhelmed by any backbone reorganizations.^66^ Given that the backbone in the CO-bound state is disordered, and that the main hydrogen bond contributor, Tyr67, does not seem to fold until later stages after photolysis, the hydrogen bond should not be the main source of stabilization for the water. In any case, the backbone contribution plays a role in the cyt *c* folding process by serving as a barrier for ligands to depart or approach the heme center. In addition to the entatic interaction that stabilized Fe–S bond in the rather static, native conformation,^5^ we have observed similar effects during the dynamic folding process that may be related to the change of functions in cyt *c*.

### Future challenges in structural reconstruction for unfolded metalloproteins

Despite the retrieval of species-associated difference signals, in the discussions above, the structural reconstruction of a heterogeneous population of unfolded metalloprotein remains a great challenge limiting the interpretation of the structural change during the folding process. TRXSS experiments provides direct kinetic information on the tertiary structural dynamics by tracking the changes in electron density in the sample as an ensemble average. For systems with well-defined ground and excited states, additional structural characterization can be employed by techniques such as rigid body modeling^67^,^68^ or shape reconstruction.^69^,^70^ However, as previously stated, in the CO-bound cyt *c* the ground and excited states represent mixtures of unfolded flexible structures requiring a higher-level modeling technique to accurately retrieve the shape data. One method to sample the unfolded ensemble is Molecular Dynamics (MD) simulations with enhanced sampling methods.^71^,^72^ Unfortunately, the results of simulations on unfolded proteins are highly sensitive to force field parameters,^73^ especially in the case of metalloproteins where metal ligation plays a determining role in the conformation of the protein.^74^ In general, force field parameters for metalloproteins remain ill-defined, and this is true even in the case of cyt *c*. Therefore, new sets of force field parameters for non-native ligations must be constructed and validated in order to reconstruct the candidate intermediate structures. In addition, the protocol to incorporate experimental data for sampling unfolded structures is under development. Our group is currently developing such force fields and protocol for MD simulations and separate works will be published in the future.

## Conclusion

We have utilized complementary XTA and TRXSS methodologies to investigate the folding of cyt *c* following CO photolysis on multiple spatial and temporal scales. Our XTA results revealed four intermediate heme ligation states, Fe*, Fe–H_2_O, Fe–Met80, and Fe–His*X*, and the structural parameters of the first three states were obtained. Our TRXSS results provide evidence for the existence of parallel conformational pathways, specifically a productive folding route through Met80 binding, and an unproductive pathway that proceeds through misligation of His*X*. Combined, XTA and TRXSS measurements revealed new structural information on folding intermediates that has not previously been revealed by optical experiments, namely the Fe–H_2_O heme ligation state before a protein residue replaces the water, the collapsed phase intermediate in Met80 binding pathway with a prolonged Fe–S distance, and the further unfolding of the protein in the His*X* binding pathway. We proposed that a local structure around heme that may spatially limit the motion of a water molecule to form the Fe–H_2_O state as well as the Met80 residue to form the Fe–Met80 state to motivate these otherwise disfavored ligations. Protected by the local structure, the Met80 remains in proximity to the heme until a later stage of folding when the bond is stabilized. We suggested that large-scale structural reorganization and loss of local structure may be the reason why some of the cyt *c* ensemble undergoes parallel folding pathways to the kinetic trap state, Fe–His*X*. Overall, the parallel experiments of XTA and TRXSS contributes to the understanding of the interplay between ligation and conformation states in metalloprotein folding dynamics by directly probing both local and tertiary structures.

## Conflicts of interest

The authors declare no competing financial interests.

## Supplementary Material

Supplementary information
