# Supplementary material for: X-ray snapshots reveal conformational influence on active site ligation during metalloprotein folding
Source: Chem Sci. 2019 Sep 3;10(42):9788–800. doi: 10.1039/c9sc02630d (PMC6993610; doi:10.1039/c9sc02630d)
Supplement: Supplementary file 1 [file SC-010-C9SC02630D-s001.pdf]

Electronic Supplementary Material (ESI) for Chemical Science.
This journal is © The Royal Society of Chemistry 2019

Supporting Information: X-ray Snapshots Reveal Conformational Influence on Active Site Ligation During Metalloprotein Folding

*Darren J. Hsu[†,‡], Denis Leshchev[†,‡], Dolev Rimmerman [†], Jiyun Hong[†], Matthew S. Kelley[†], Irina Kosheleva[∥], Xiaoyi Zhang[⊥], Lin X. Chen[†,§,]**

[†]Department of Chemistry, Northwestern University, Evanston, Illinois 60208, USA

[∥]Center for Advanced Radiation Sources, The University of Chicago, Illinois 60637, USA

[⊥]X-ray Sciences Division of the Advanced Photon Source and [§]Chemical Sciences and Engineering Division, Argonne National Laboratory, Argonne, Illinois 60439, USA

‡ These authors contributed equally to this work

*To whom correspondences should be addressed

Supporting Information

SI.1 Experimental setup

XTA Experimental setup

The pump pulses were 560 µm x 340 µm (W x H, fwhm). The X-ray probe pulses were 500 µm x 200 µm (W x H, fwhm) centered at the laser pump. The pump / probe pulses were aligned on a flowing cylindrical jet with a ~ 600 µm diameter. For XANES region (up to 7212 eV), signal was integrated for 2 seconds for each energy point. For EXAFS region (above 7212 eV), signal was integrated longer (at least 4 seconds and as a function of incident X-ray energy) to achieve sufficient signal-to-noise ratio. An iron foil was placed downstream for energy calibration. The X-ray fluorescence signal as a function of incident X-ray energy was collected with two avalanche photodiodes (APDs) perpendicular to both the jet and the X-ray direction on both sides, facing the jet. The third APD detector was placed upstream to record incident X-ray flux.

TRXSS experimental setup

TRXSS experiments were carried out at BioCARS 14-ID-B beamline at the Advanced Photon Source (APS). Details of the X-ray scattering setup and generic TRXSS pump-probe data acquisition methodology at BioCARS have been previously published.[1–3] The sample was loaded into a temperature controlled capillary flow cell set at 25 °C. During data collection the capillary was translated by 200 micron steps over a range of ~5 cm (i.e, ~250 data collection spots) such that each spot on the capillary was only probed by X-ray/laser pairs once in order to mitigate X-ray and laser damage. In addition, the sample in the capillary was refreshed after each full translation of the capillary by utilizing a syringe pump in order to further mitigate any effects of damage. For pump-probe measurements, the sample was excited by 7 ns laser pulses with 532 nm wavelength to initiate CO photolysis and then probed by electronically delayed X-ray pulses. The laser beam was focused on a capillary with the sample solution to an elliptical spot 100 µm x 450 µm delivering energy of ~1-2 mJ in each pulse. The X-ray pulses, comprised of a pink spectrum centered at 11.65 keV, were focused by a pair of K-B mirrors to a beamsize of 40 µm by 65 µm at the sample.

The chopper-shutter system described in literature[2] was employed to reduce the repetition rate of the synchrotron source operating in 24 bunch mode. The chopper opening time was used to modify the time resolution of the experiment at the cost of X-ray flux. The chopper opening time was calibrated to 3.7 µs for long time delays (longer than 5 µs). Similarly, for short time delays (delays shorter than 5 µs) the opening time was ~500 ns. The repetition rate of the experiment was adjusted from 5 Hz to 1.5 Hz depending on the investigated time delay range. Data collection was set up to collect a time series of specific positive time delays spanning from nanoseconds to milliseconds, interwoven with negative time delays to allow for calculation of difference curves. The positive time delays in the data collection series were rotated randomly to verify that there were no erroneous signals.

SI.2 XTA data preprocessing

Energy calibration. After data collection, energy calibration was done through the iron foil downstream of the X-ray path. Recorded energy was offset by aligning the edge of the iron foil absorption spectrum to 7112 eV.

Creation of raw dataset. Signal from the two APDs facing the jet were averaged and normalized with respect to X-ray flux recorded with the upstream APD. Signal from each of scan was inspected and apparent outliers due to unstable jet (low counts) and damaged sample (high background) were discarded. 79 scans were selected for further analysis.

Difference spectra. We first construct the basis spectra of the ground state. All pre-laser (600) bunches were averaged as the true ground state Fe-CO spectrum. Each pre-laser bunch was subtracted by this spectrum using least squares approach, which left structured noises clearly due to different integration times used in EXAFS region. (**Figure S 1**) Singular value decomposition (SVD) analysis was further performed on the residual noise, which revealed two significant components. To obtain difference spectrum for each post-laser bunch, the raw spectrum was subtracted by the true ground state spectrum and two noise spectra again with the least squares approach. The residue was treated as the difference signal.

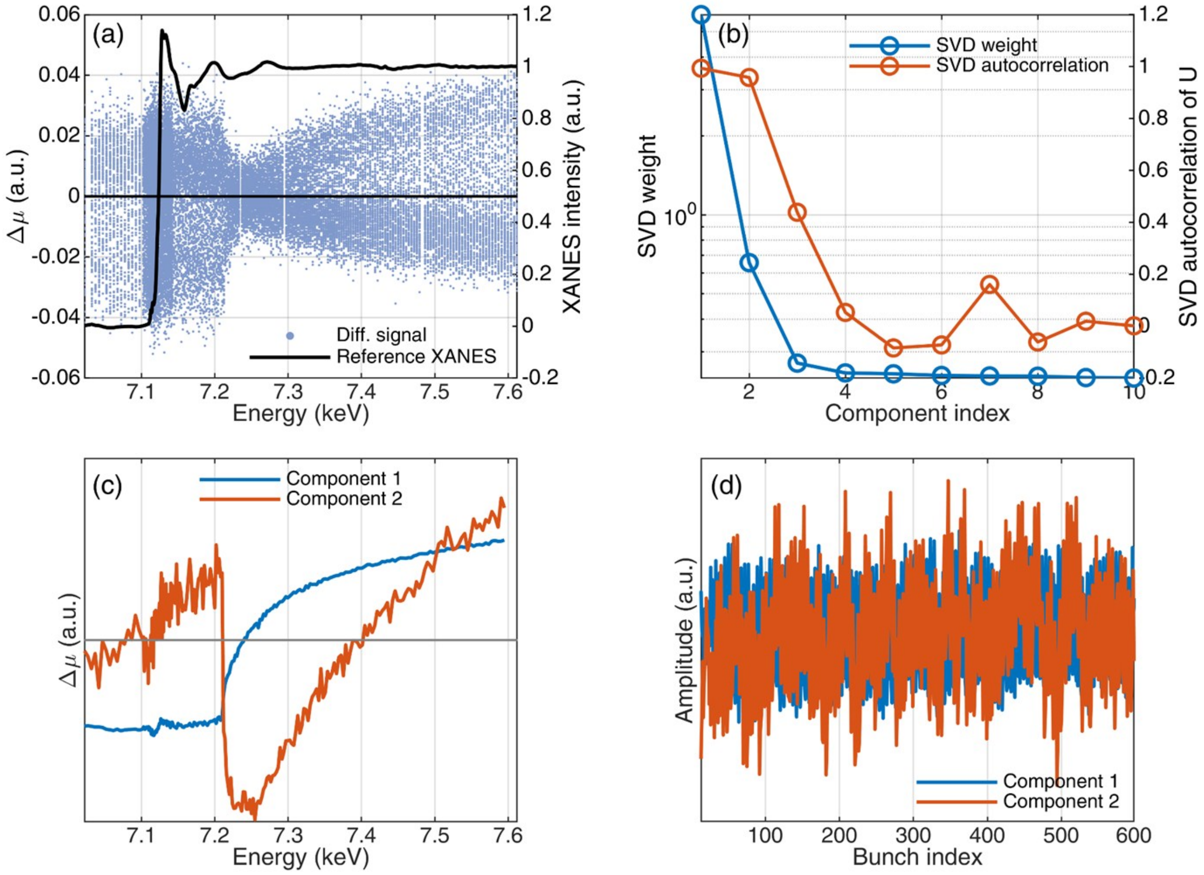

Figure S 1. Singular value decomposition (SVD) of residual noise. Residual noise was obtained by subtract raw signal from each bunch with the averaged raw signal of the ground state. **(a)** Plot of the raw noise (blue dots).

A normalized XANES spectrum (black, scale to the right axis) is included for comparison. **(b)** SVD weights (blue, left axis) and autocorrelation (red, right axis) of each component. **(c)** SVD spectral components. **(d)** SVD kinetic traces of the two components in (c).

SI.3 XTA difference data analysis and global analysis (GA)

As stated in main text, the earliest 1 ns time delay was separated from the rest for further analysis since the timescales differ by roughly two orders of magnitude and that the water could not have entered the heme pocket at 1 ns. The signal at 1 ns is distinctively different from the next bunches (t = 154 and 307 ns). (**Figure S 2**) SVD of the rest (from 154 ns on) of the difference data revealed two significant components with autocorrelation values > 0.8 and three relevant timescales, about 2 μs, 20 μs, and 200 μs (**Figure S 3**). The autocorrelation function is calculated as $\sum_j U(E_j)U(E_{j+1})$ for each left singular vector $U(E)$ as a function of energy E. A threshold of 0.8 is used to distinguish signal from noise. However, in SVD analysis each component contains a mix of signal from all timescales, making it difficult to interpret the result. Global analysis (GA) was then employed to separate the two SVD components to species-associated signals with corresponding kinetic trace that reflects meaningful physical processes.

Following **Scheme 1** in main text, the two processes (Fe-H_2O to Fe-Met80 and to Fe-HisX) are modeled to be parallel and single exponential, with respective populations A_M and $1 - A_M$ and time constants k_M and k_H. At later time delays, the signal decays approximately linearly due to the laser excited volume exiting the X-ray probe volume with a (zeroth-order) time constant k_X. The associated equations for the populations of each species can be expressed as:

$$[Fe - H_2O](t) = A_M \exp(-k_M t) + (1 - A_M)\exp(-k_H t)$$

$$[Fe - HisX](t) = (1 - A_M)(1 - \exp(-k_H t)) - k_X t$$

$$[Fe - Met80](t) = A_M(1 - \exp(-k_M t)) - k_X t \ .$$

Since only two SVD components were significant, we first modeled the two (signal) states to be Initial and Fe-L (L = HisX and Met80) states to isolate the state-associated difference spectra in early time delays.

$$[Initial](t) = A_M \exp(-k_M t) + (1 - A_M)\exp(-k_H t)$$

$$[Fe - L](t) = (1 - A_M)(1 - \exp(-k_H t)) + A_M(1 - \exp(-k_M t)) - 2k_X t$$

The two decay time constants of the Initial signal state for the best fit were $k_M = 1.6 \pm 0.33$ μs and $k_H = 15 \pm 8.0$ μs, with $A_M = 0.82 \pm 0.056$. (**Figure S 4a,b** and **Table S 1**)

Comparing the Initial signal state from GA with the simulated XANES difference spectra suggests that the signal is associated with the Fe-H_2O ligation (**Figure S 5a** and **Figure S 5b**). Comparing the Heme-L signal state to the simulated Fe-HisX and Fe-Met80 difference spectra revealed that the latter is a better fit (**Figure S 5c**). While the Fe-L signal should be a combination of Fe-HisX and Fe-Met80 signals, the latter being a better fit can be explained by that the simulated XANES difference spectra for the Fe-H_2O and Fe-HisX states turned

out to be very similar. (**Figure S 6**) Therefore, the evolution between these two species will not generate much signal. Indeed, when the state representation was changed to that state 1 = Fe-H_2O + Fe-HisX, and state 2 = Fe-Met80, the state-associated difference spectra, kinetic parameters and respective weights were almost identical (**Table S 1** and **Figure S 4c,d**), again showing the signal generated from the transition from the Fe-H_2O state to the Fe-HisX state is very weak. The formation time constant for the Fe-Met80 state, 2.1 ± 0.24 μs in this representation, matches the value obtained in the OTA studies, corroborating the assignment. This finding also allowed us to extract the species-associated difference spectrum for the Fe-Met80 state (**Figure 3a**, bottom row in main text) and plot the kinetic trace of each species (**Figure S 5d**).

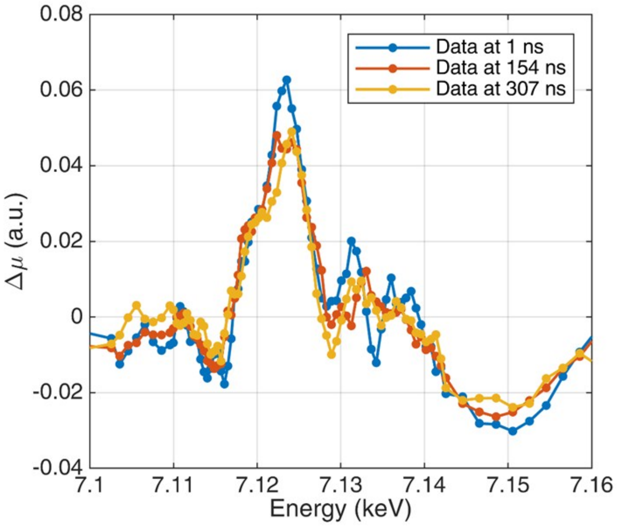

Figure S 2. Comparison between the difference spectrum of first X-ray bunch (sync bunch, t = 1 ns) after laser pulse (blue) and those of 2 subsequent bunches (t = 154 ns and 307 ns, red and yellow, respectively). Curves were three-point smoothed for clarity.

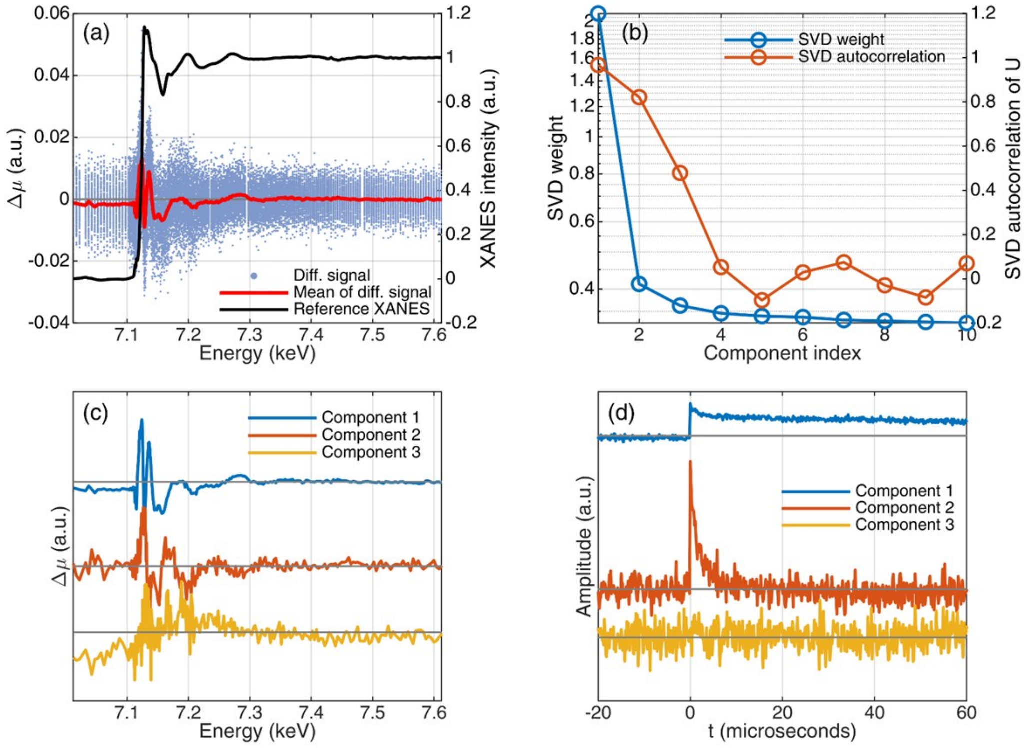

Figure S 3. SVD of difference signal. **(a)** Plot of the raw difference signal (blue dots) and the average (red). A normalized XANES spectrum (black, scale to the right axis) is included for comparison. **(b)** SVD weights (blue, left axis) and autocorrelation (red, right axis) of each component. **(c)** SVD spectral components. **(d)** SVD kinetic traces of the two components in (c). Only the first two components have meaningful kinetic traces beyond noise.

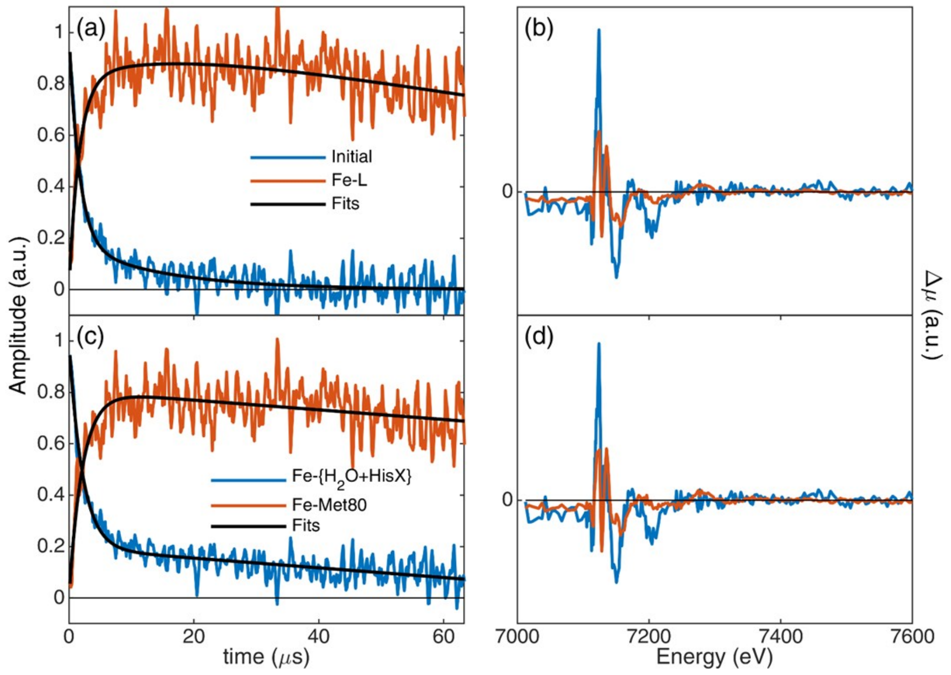

Figure S 4. Global analysis (GA) results with two different state representations. The two models yielded similar results, indicating that Fe-HisX signal does not differ much from Fe-H_2O signal. **(a)** State evolution with one (decay-only) Initial state (blue) and Fe-L state (red). Traces were three-point averaged in time. **(b)** State-associated difference spectra of (a). Line colors correspond to that in (a). **(c)** State evolution with State 1 = Fe-H_2O + Fe-HisX (blue) and State 2 = Fe-Met80 (red). Traces were three-point averaged in time. **(d)** State-associated difference spectra of (c). Line colors correspond to that in (c).

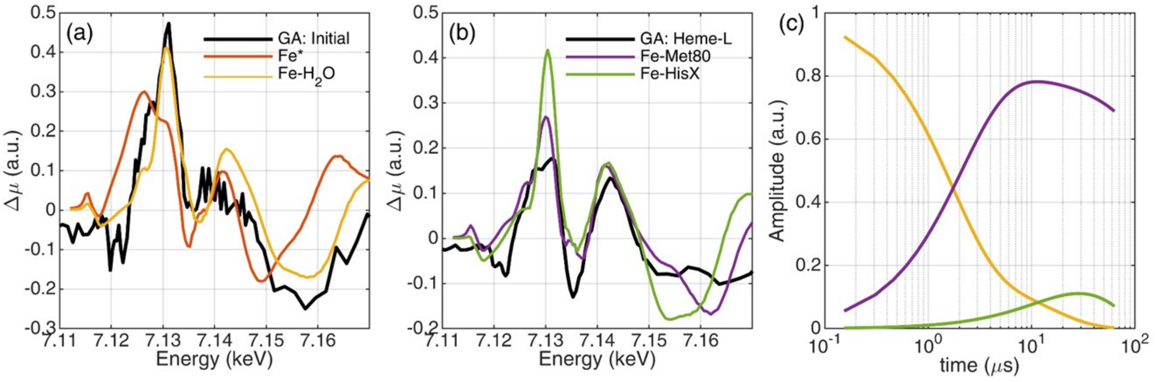

Figure S 5. Species assignment for XTA. **(a, b)** Comparison between state-associated (black) and simulated (colored) XANES difference spectra using DFT minimized structures. **(c)** Kinetic trace for each species. The Fe* state is assumed to have evolved to the Fe-H_2O state at 154 ns time delay.

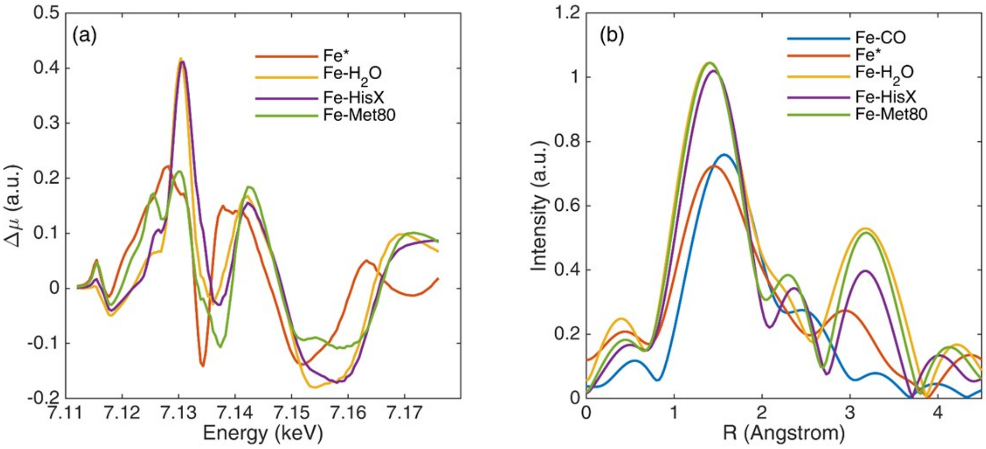

Figure S 6. (a) Comparison of simulated XANES difference spectra relative to ground state (Fe-CO). **(b)** Comparison of simulated Fourier Transformed EXAFS R-space distribution. Both regions combined, bis-his state and Fe-H_2O states are similar and evolution between the two species will not generate much signal.

Table S 1. Kinetic parameters after fitting XTA difference data with global analysis using two different models.

	**Model 1**	**Model 2**
State 1	Fe-H_2O	Fe-H_2O + Fe-HisX
State 2	Fe-HisX + Fe-Met80	Fe-Met80
A_H	0.18 ± 0.056	0.19 ± 0.17
A_M	0.82 ± 0.056	0.81 ± 0.17
$k_H(\mu s^{-1})$	15 ± 8.0	N/A†
$k_M(\mu s^{-1})$	1.6 ± 0.33	2.1 ± 0.24
$k_X(\mu s^{-1})$	264 ± 28	265 ± 29

†In this representation Fe-H_2O to Fe-HisX transition shows up as a constant signal in State 1, and k_H is not a parameter.

SI.4 Simulated XANES difference spectra

The simulated XANES difference spectra we utilized in the main text to help assigning states were calculated with FEFF9.6.[4] The base input structure was taken from crystal structure of 1HRC comprising the heme, Cys14, Cys17, and Met80. For Fe-CO state we replaced Met80 with a CO molecule perpendicular to the heme ring. For Fe* and Fe-H_2O state structures, we performed Density Functional Theory optimization (B3LYP functional and 6-31G* basis sets) after replacing Met80 with corresponding structures. For Fe-Met80 state we simply took

the 1HRC structure. The structures were input into FEFF and the spectra were calculated with XANES card with 4.5 Å self-consistent field. S_0^2 was set to 0.85. The difference spectra were calculated by subtracting Fe-CO spectrum from other states. We then shifted the difference spectra in energy and amplitude to match each species-associated difference spectrum from GA with least square method. We made the state assignments based both on this approach and the kinetic information from the previous studies. After the EXAFS fitting, we recalculated the XANES difference spectra with the determined Fe-O and Fe-S bond lengths (2.34 Å and 2.65 Å, respectively). Both difference spectra still match the species-associated difference spectra, corroborating our assignments (**Figure S 7**).

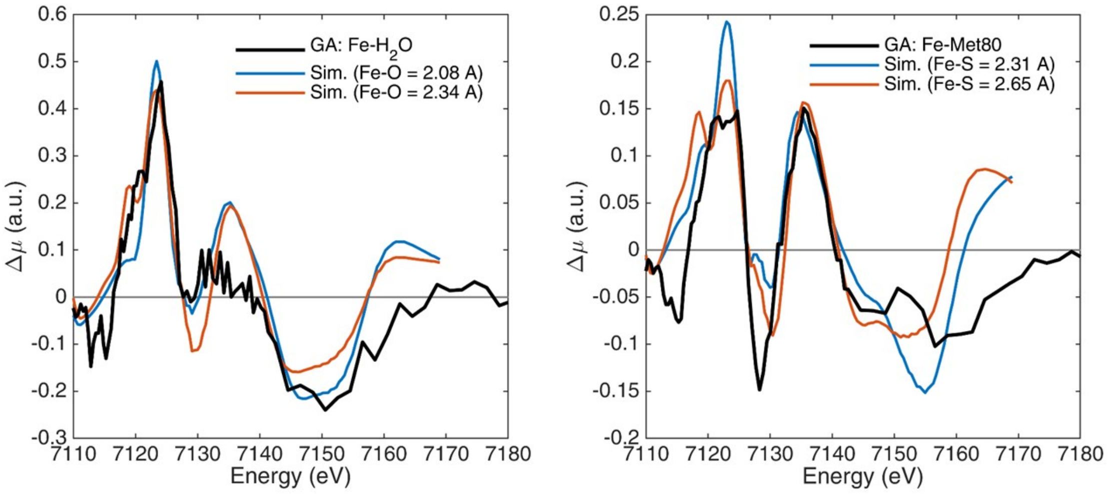

Figure S 7. Comparing simulated difference spectra before and after EXAFS fitting with different lengths of the iron to ligating atoms. (Left) Fe-H_2O state (Right) Fe-Met80 state. GA, difference spectrum from global analysis (i.e. species-associated difference spectrum). Sim., Simulated difference spectrum.

SI.5 Excited state fraction and reconstructed spectra

Excited state fraction was determined the intensity loss of the second pre-edge peak unique to Fe-CO spectrum. An exponential baseline fitted from 7.063 to 7.122 keV excluding 7.111 to 7.118 keV (where pre-edge features are) was applied to isolate the pre-edge peaks. The two peaks were deconvoluted with two Gaussians. The process is shown in **Figure S 8**. First 60 bunches (9.2 µs) after laser pulse were averaged to give an estimated excitation fraction of ~0.49. Since post-laser bunches were over-subtracted by full ground state spectrum (by 1 / excited state fraction), the species-associated difference spectra obtained this way must be scaled up by 1 / excited state fraction to properly reconstruct the species-associated absolute spectra for EXAFS analysis.

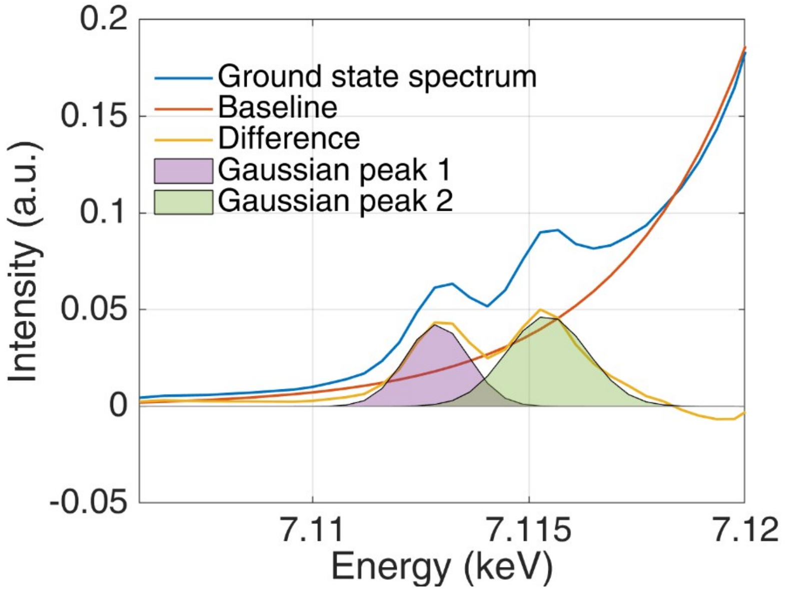

Figure S 8. Determining excited state fraction. Ground state spectrum (blue) is subtracted by an exponential rising background (red), and the difference (yellow) is fit with two Gaussian peaks (purple and green). Only the second peak (green) is utilized in determination of excited state fraction.

SI.6 k-space EXAFS signal

The signal of k-space EXAFS is a sum of Fourier Transformation of distance function, which means a change in bond distance (resembling a peak shift in R-space EXAFS) will be transformed into an oscillation spread out in the entire k-space. The change therefore may not be immediately obvious. **Figure S 9** shows k-space EXAFS signals from all four states overlaid. The signals from Fe-H_2O and Fe* are particularly hard to distinguish. The main differences between the two spectra are at 3.5 $Å^{-1}$ where the intensity is different and at 4.5 $Å^{-1}$ and 6 $Å^{-1}$ where slight red shifts of Fe-H_2O signal can be found.

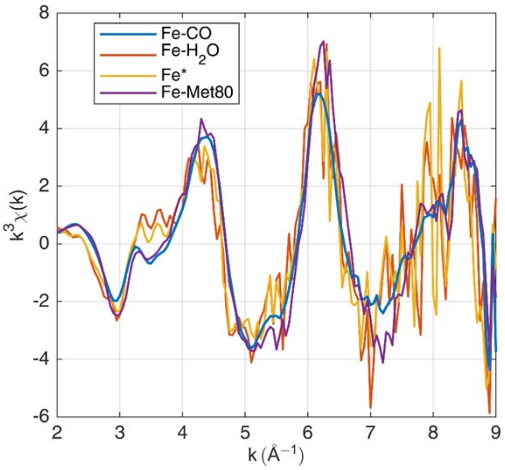

Figure S 9. k-space EXAFS spectra of the identified species overlaid to show subtle differences.

SI.7 EXAFS analysis

To gain insight into active site structural dynamics, multiple scattering EXAFS analysis was carried out for the ground state and intermediate states using their species-associated EXAFS spectra. The modulation of absorption spectra $\chi(k)$ as a function of electron wavevector k can be modeled with the following equation,[5–7]

$$\chi(k) = \sum_i F_i(k) S_0^2(k) N_i / \left(kR_i^2\right) \exp\left(-2\sigma_i^2 k^2\right) \sin\left[2kR_i + \phi_i(k)\right]$$

where the summation is over i paths, $F(k)$ the magnitude of backscattering, S_0^2 the factor of amplitude reduction, N the coordination number, R the path length, σ^2 mean-squared displacement, and Φ the phase shift. Analysis was performed with Athena and Artemis packages based on FEFF software.[4,8] The general model and structure utilized for fitting are shown in **Scheme 2** in the main text. The crystal structure (PDB entry: 1HRC[9]) was used as initial coordinates. For Fe-CO state, Met80 was replaced by CO perpendicular to the heme plane with a Fe-C bond distance of 1.75 Å.[10–12] Similarly, for Fe-H_2O state an oxygen atom was placed at 2.25 Å from heme iron. To reduce the numbers of fitting parameters, the 5 Fe-N coordinations from the porphyrin and His18 were considered the same type. Atoms further than the first shell were grouped by distance to the iron center (C_α, C_m, and C_β), and nitrogen atoms were not distinguished from carbon atoms. Therefore, using the notation in the 1HRC pdb file, the CE1 and CD2 atoms on His18 were treated as "C_α", CG and ND1 as "C_β". CE and CG atoms on Met80 were grouped into "C_m", and CB into "C_β".

Precision of atom pair distance estimation. In the EXAFS fits with ARTEMIS, the reported uncertainties (1 sigma) of the atom pair distances are smaller than 0.01 Å for Fe-N. For all other atom pairs, the uncertainty of distance is in the range between 0.01 to 0.03 Å.

SI.8 TRXSS solvent subtraction

The protein associated TRXSS signals were obtained via standard procedure of solvent heating subtraction from TRXSS signal as described before.[3] The heating signal was measured by exciting the 6 mg/ml solution of oxidized cyt *c* with 527 nm laser pulses and recording TRXSS data at 10 μs. Since oxidized cyt *c* does not undergo any structural transitions upon excitation of the heme Q band, the difference TRXSS signal obtained in such way includes only solvent heating data. The recorded heating signal was then fit to the CO-bound cyt *c* data in the q-range between 0.9 $Å^{-1}$ and 2.0 $Å^{-1}$. The results of the solvent heating contribution subtraction are shown in **Figure S 10** for both early and late time delay data sets.

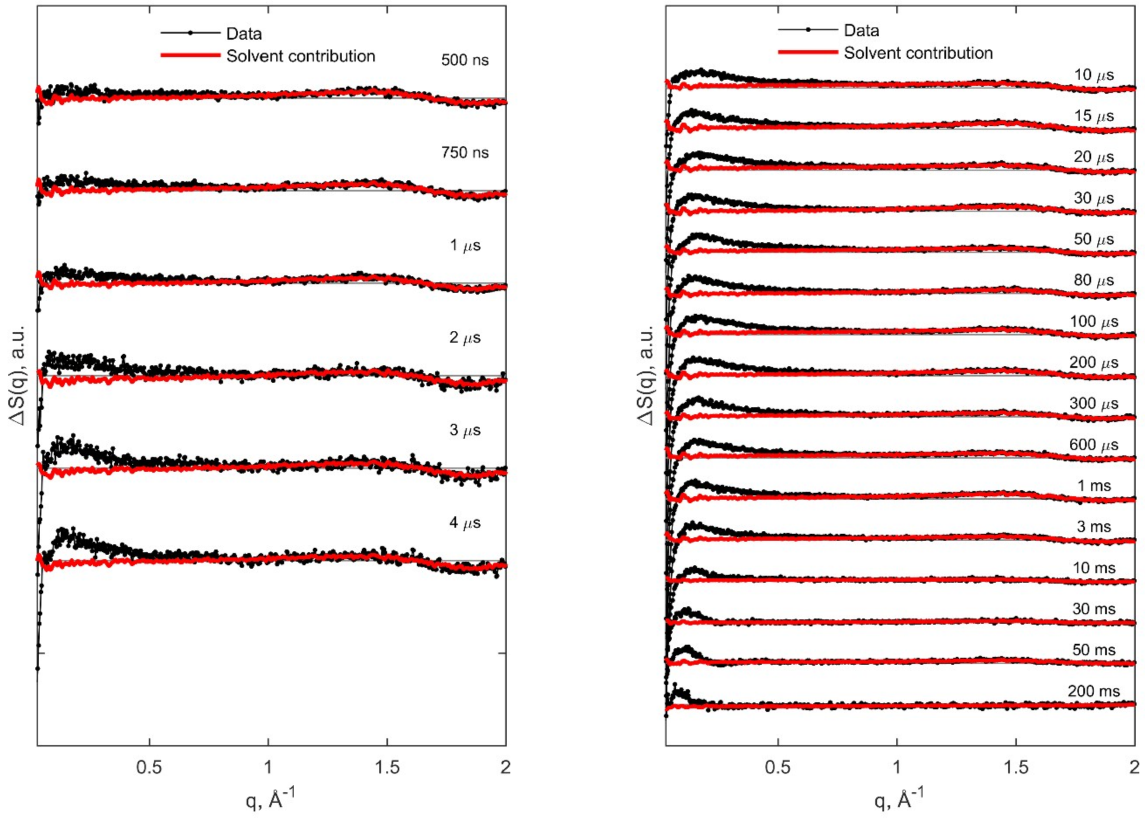

Figure S 10. Subtraction of solvent contribution from the CO-bound cyt *c* data. **(Left)** Solvent contribution (red) and CO-bound cyt *c* data (black) recorded at early time delays (t ≤ 4 μs) using 18 mg/mL protein concentration. **(Right)** The same as left panel, but for later time delays (t ≥ 10 μs) that were recorded using 6 mg/mL protein concentration.

SI.9 GA of TRXSS data

The global analysis was performed on the protein-associated TRXSS data using standard procedures[3,13–15] with the aid of Singular Value Decomposition (SVD). GA was performed separately on the data recorded at early time delays (t ≤ 4 μs) and on the data recorded at later time delays (t ≥ 10 μs). GA was performed using kinetic model described in the text.

GA of the early time delay data (t ≤ 4 μs) is shown in **Figure S 11**. Inspection of the singular values indicates that the first singular value has substantially larger magnitude compared to the others. Additionally, the autocorrelation (AC_i) calculated for all left singular vectors $U_i(q)$ as

$$AC_i = \sum_j U_i(q_j) U_i(q_{j+1})$$

shows that only the first singular value has AC value larger 0.8 – a typical threshold chosen to distinguish between SVD components containing signal and the noise – indicating that only the first SVD component is necessary to describe the data. The protein associated TRXSS data is then globally fit with the first left singular vector $U_i(q)$ and a single exponential rise to represent the emerging of the protein conformational state denoted as U_M in the main text. The time scale of the formation of the U_M was determined to be $\tau_M = 1.8 \pm 0.1\ \mu s$.

GA of the later time delay data (t ≥ 10 μs) is shown in **Figure S 12**. Inspection of the singular values and the AC values indicates that 3 components are necessary to describe the data set. The fitting was performed using the following kinetic equations (see **Scheme 1** in the main text):

$$C_{U_M}(t) = \exp\left(-\frac{t}{\tau_X}\right)$$

$$C_{F_M}(t) = 1 - \exp\left(-\frac{t}{\tau_X}\right)$$

$$C_{U_H}(t) = \exp\left(-\frac{t}{\tau_Z}\right) - \alpha\exp\left(-\frac{t}{\tau_{Y1}}\right) - (1-\alpha)\exp\left(-\frac{t}{\tau_{Y2}}\right)$$

with the $C_{U_M}(t)$, $C_{F_M}(t)$, and $C_{U_H}(t)$ being concentrations of the U_M, F_M, and U_H species, respectively (see main text for the assignment). The best fit time scales are as following: $\tau_X = 6.6 \pm 0.7\ ms$, $\tau_Z = 20.4 \pm 0.7\ ms$, $\tau_{Y1} = 18 \pm 1\ \mu s$, and $\tau_{Y2} = 400 \pm 40\ \mu s$; the best fit partition coefficient $\alpha = 0.66 \pm 0.01$, that describes the partitioning between fast and slow components of the His26/33 binding. The kinetic parameters obtained from GA are summarized in **Table S 2**.

We note that the present kinetic model does not take into account the CO rebinding of cyt *c* while in U_M state, which would result in an additional decay of the U_M species to the ground state CO-bound cyt *c*. While we attempted to include this decay into GA of long time scale data (t ≥ 10 μs), the statistical fitting quality (χ^2 metric) improvement was negligible, making such additional term unnecessary for sufficient fitting.

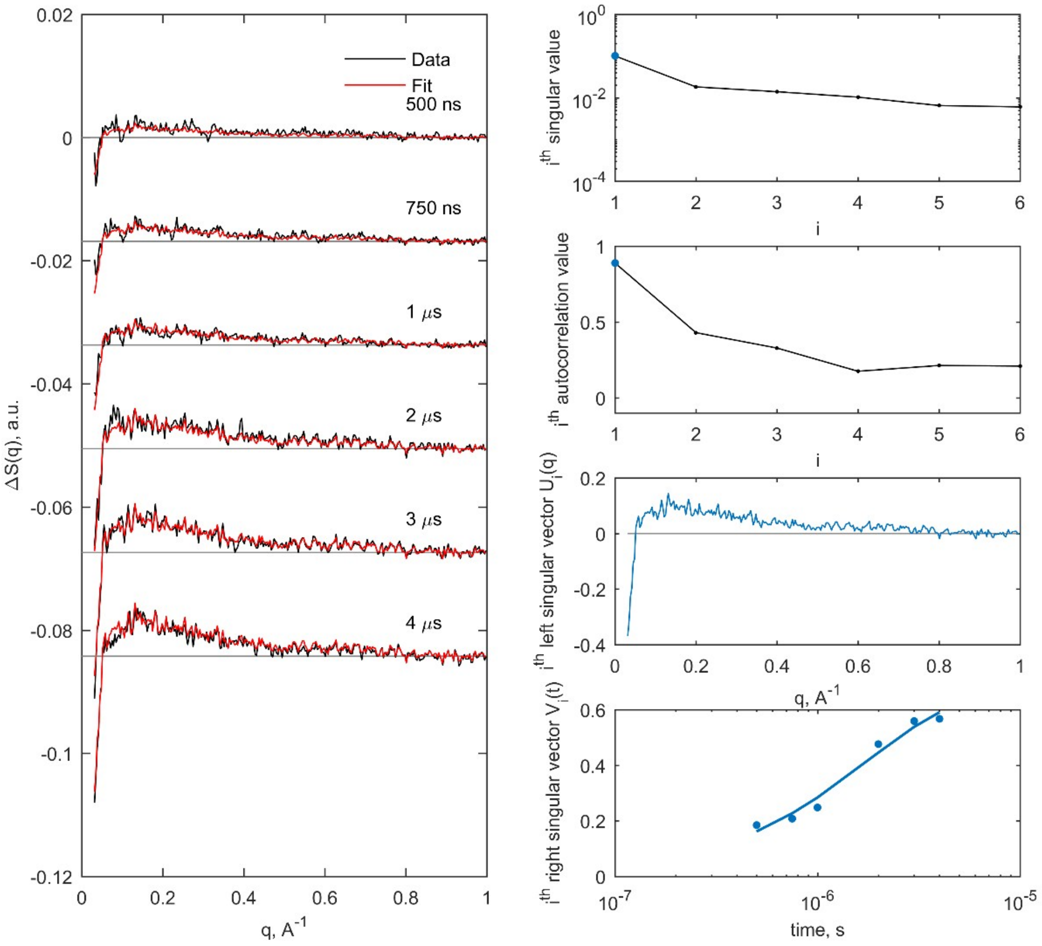

Figure S 11. GA of the protein associated TRXSS data recorded at short time delays (t ≤ 4 μs). **(Left)** Comparison of the data and the GA fit. **(Right)** From top to bottom: singular values, autocorrelation values, left singular vectors, right singular vectors (circles) and the fit (line). Singular vectors, values, and autocorrelation values have the same color code throughout the left panels.

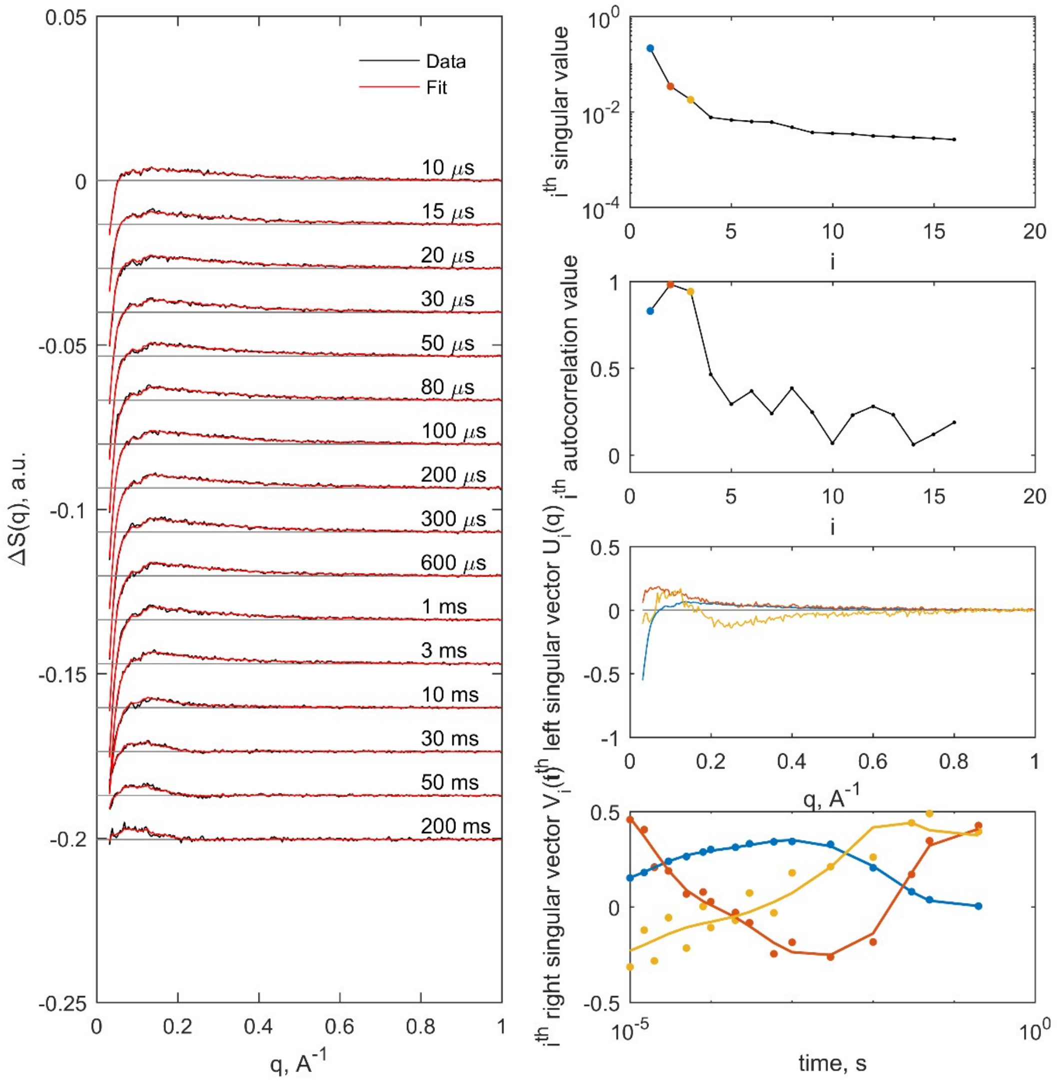

Figure S 12. GA of the protein associated TRXSS data recorded at long time delays (t ≥ 10 μs). **(Left)** Comparison of the data and the GA fit. **(Right)** from top to bottom: singular values, autocorrelation values, left singular vectors, right singular vectors (circles) and the fit (line). Singular vectors, values, and autocorrelation values have the same color code throughout the left panels.

Table S 2. Kinetic parameters after fitting TRXSS difference data with global analysis. Terms are defined in SI text.

Parameters	Value(s)
$\tau_M(\mu s)$	1.8 ± 0.11
$\tau_X\ (ms)$	6.6 ± 0.69
$\tau_{Y1}(\mu s)$	18 ± 1.1
$\tau_{Y2}(\mu s)$	400 ± 37
$\tau_Z(ms)$	20 ± 0.68
A_{U_H}	0.66 ± 0.011

SI.10 Guinier analysis

To obtain the insight into the changes in the size of the protein, we performed Guinier analysis of the scattering data. For the static data, the fitting was done using standard procedure. The static scattering data for CO-bound cyt *c* (U_{CO}) and native, folded (F_M) species, as well as the Guinier fitting results, are shown in **Figure S 13**. The best fit values of the radii of gyration R_g for unfolded CO-bound and folded cyt *c* are 24.6 ± 0.4 Å and 13.6 ± 0.3 Å, respectively.

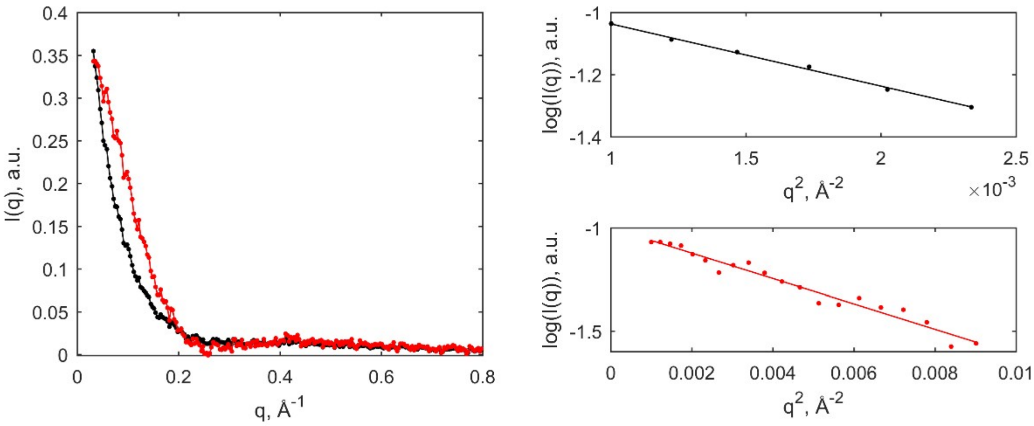

Figure S 13. Static scattering curves for cyt *c* and their Guinier fitting. **(Left)** Static curves for CO-bound cyt *c* (black) and native cyt *c* (red). **(Right)** Guinier fit of the static data for (top) CO-bound and (bottom) native cyt *c*.

To extract the radii of gyration for the transient species, we fitted the difference patterns using Guinier approximation in the low q region of the TRXSS data, as was done before in the literature.[16] To fit the Guinier equation to the difference signal we have used the following equation:

$$\Delta S(q) = \alpha\left(I_0 \exp\left(-\frac{q^2 R_g^2}{3}\right) - I_{U_{CO}}(q)\right)$$

where $I_{U_{CO}}(q)$ is the experimentally measured static scattering curve for U_{CO} species (the ground state), I_0 and R_g are the forward scattering intensity and the radius of gyration of cyt *c* in an intermediate state, and α is the scaling factor. To verify that the procedure provides a reliable estimation of R_g, we tested the performance of this fitting procedure on the difference TRXSS signal for F_M species (**Figure S 14**). The fitting resulted in R_g being 12.5 ± 1.5 Å, which is in agreement with the standard Guinier fitting procedure implemented for the total (i.e. not difference) static data. Fitting the TRXSS signal for cyt *c* in U_M state is shown in **Figure S 14**, and results in R_g = 18.2 ± 1.2 Å.

We note that U_H signal is comprised of a significant loss of the intensity in the low-q region, which indicates further unfolding of the protein, as discussed in the main text. Larger size of the protein prevents using the Guinier approximation, as the number of points in the low q region becomes too small for a reliable estimation of the protein size in the intermediate state.

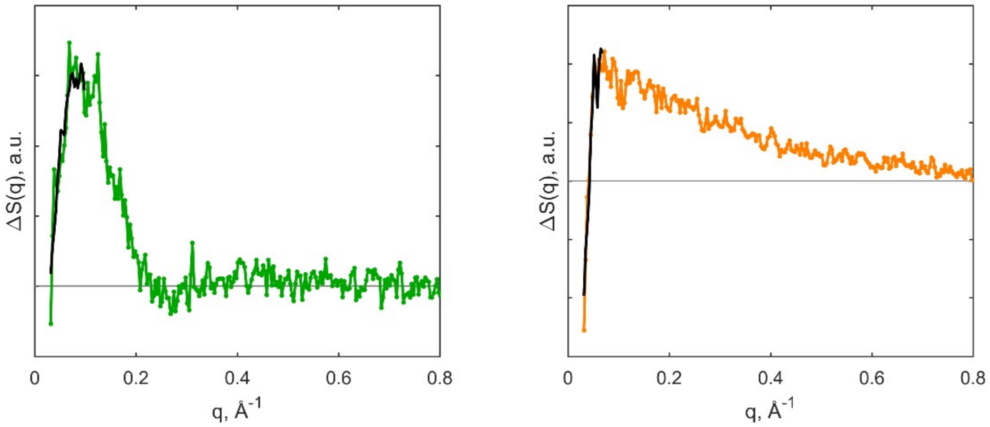

Figure S 14. Guinier fitting of the difference TRXSS signals for F_M (left) and U_M (right) conformational states. The data are shown in green and orange and the fits are shown in black.

SI.11 Bayesian Inverse Fourier Transform (BIFT) analysis

To gain a more intuitive insight into the difference scattering patterns, we investigated the scattering data in the real space by calculating pair distribution functions using BIFT with the smoothing constraint.[17–19] The results for both static data and the difference are shown in **Figure S 15**. From the comparison between pair distribution functions $P(r)$ for F_M and U_{CO} species, it follows that the electron density in the unfolded state is spread over a larger space compared to the folded conformation. Further analysis of the difference TRXSS signals for each species provides insight about associated structural changes in the protein as compared to the ground state U_{CO} conformation. For U_M, we observe a strong gain in the electron density at distances $r < 15$ Å, which indicates

the possible formation of the secondary structure; the loss of electron density at larger distances $r > 15$ Å, which accompanies the positive peak at shorter distances, indicates that the protein becomes more compact, which is in agreement with the Guinier analysis. In case of difference TRXSS signal for F_M, we observe a gain in electron density at $r < 30$ Å indicating a much stronger structural reorganization associated with the folding. $P(r)$ obtained from TRXSS curve and the static difference agree well at $r < 40$ Å and slightly diverge at larger distances. The difference between the TRXSS and static curves indicates the uncertainty associated with the TRXSS species associated differences. Finally, the U_H curve exhibits a loss of electron density spanning from the r values up to the maximum size of cyt *c* in U_{CO} conformation, which is approximately 90 Å (see figure X). We note that in the present experiments the lowest available q value was 0.03 Å^{-1}, which makes the measurement sensitive only up to distances $\frac{\pi}{q_{min}} \sim 105$ Å (vertical grey line in **Figure S 16**). Therefore, while the loss of electron density up to 105 Å for U_H indicates further unfolding compared to the U_{CO} conformation, the structural information about the degree of such unfolding is not available from the present data. The negative signal observed at $r > 105$ Å is not a reliable representation of structural rearrangement of cyt *c* in U_H conformation.

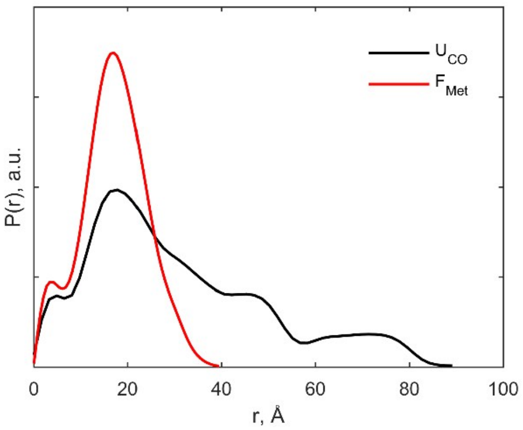

Figure S 15. Comparison of pair distribution functions P(r) for UCO and FM conformations of cyt *c* obtained using BIFT of the static data.

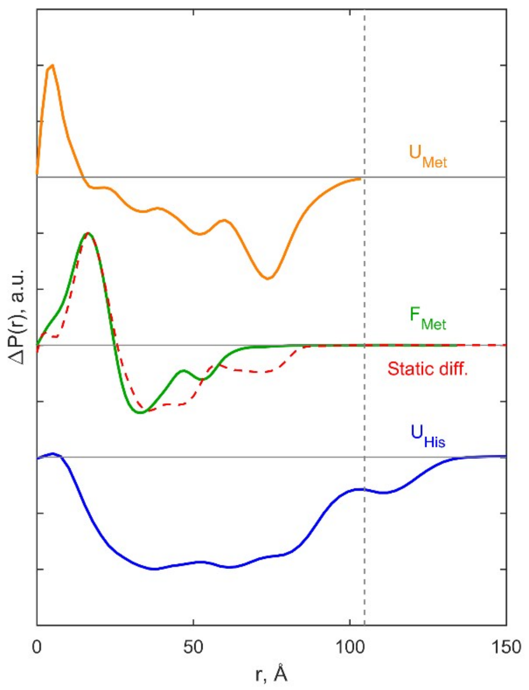

Figure S 16. Comparison of pair distribution functions $P(r)$ for U_M, F_M, and U_H conformations of cyt *c* obtained using BIFT of the species associated difference TRXSS curves. Orange – U_M, green – F_M, red dashed – static difference, blue - U_H. The vertical dashed grey line shows the maximum distance up until which the calculated $P(r)$ are reliable.

References

1 M. Cammarata, M. Levantino, F. Schotte, P. a Anfinrud, F. Ewald, J. Choi, A. Cupane, M. Wulff and H. Ihee, *Nat. Methods*, 2008, **5**, 881–886.

2 T. Graber, S. Anderson, H. Brewer, Y.-S. S. Chen, H. S. Cho, N. Dashdorj, R. W. Henning, I. Kosheleva, G. Macha, M. Meron, R. Pahl, Z. Ren, S. Ruan, F. Schotte, V. Šrajer, P. J. Viccaro, F. Westferro, P. Anfinrud and K. Moffat, *J. Synchrotron Radiat.*, 2011, **18**, 658–670.

3 D. Rimmerman, D. Leshchev, D. J. Hsu, J. Hong, I. Kosheleva and L. X. Chen, *J. Phys. Chem. Lett.*, 2017, **8**, 4413–4418.

4 J. J. Rehr, J. J. Kas, F. D. Vila, M. P. Prange and K. Jorissen, *Phys. Chem. Chem. Phys.*, 2010, **12**, 5503–5513.

5 D. E. Sayers, E. A. Stern and F. W. Lytle, *Phys. Rev. Lett.*, 1971, **27**, 1204–1207.

6 F. W. Lytle, D. E. Sayers and E. A. Stern, *Phys. Rev. B*, 1975, **11**, 4825–4835.

7 E. A. Stern, D. E. Sayers and F. W. Lytle, *Phys. Rev. B*, 1975, **11**, 4836–4846.

8 B. Ravel and M. Newville, *J. Synchrotron Radiat.*, 2005, **12**, 537–541.

9 G. W. Bushnell, G. V. Louie and G. D. Brayer, *J. Mol. Biol.*, 1990, **214**, 585–595.

10 Y. Zhang and E. A. Stern, *Phys. B Condens. Matter*, 1995, **208–209**, 727–728.

11 M. W. Mara, M. Shelby, A. Stickrath, M. Harpham, J. Huang, X. Zhang, B. M. Hoffman and L. X. Chen, *J. Phys. Chem. B*, 2013, **117**, 14089–14098.

12 A. B. Stickrath, M. W. Mara, J. V. Lockard, M. R. Harpham, J. Huang, X. Zhang, K. Attenkofer and L. X. Chen, *J. Phys. Chem. B*, 2013, **117**, 4705–4712.

13 D. Rimmerman, D. Leshchev, D. J. Hsu, J. Hong, B. Abraham, R. Henning, I. Kosheleva and L. X. Chen, *J. Phys. Chem. B*, 2018, **122**, 5218–5224.

14 D. Rimmerman, D. Leshchev, D. J. Hsu, J. Hong, B. Abraham, I. Kosheleva, R. Henning and L. X. Chen, *Photochem. Photobiol. Sci.*, 2018, **17**, 874–882.

15 D. Rimmerman, D. Leshchev, D. J. Hsu, J. Hong, B. Abraham, R. Henning, I. Kosheleva and L. X. Chen, *J. Phys. Chem. B*, 2019, **123**, 2016–2021.

16 M. Levantino, G. Schirò, H. T. Lemke, G. Cottone, J. M. Glownia, D. Zhu, M. Chollet, H. Ihee, A. Cupane and M. Cammarata, *Nat. Commun.*, 2015, **6**, 6772.

17 S. Hansen, *J. Appl. Crystallogr.*, 2000, **33**, 1415–1421.

18 B. Vestergaard and S. Hansen, *J. Appl. Crystallogr.*, 2006, **39**, 797–804.

19 A. H. Larsen, L. Arleth and S. Hansen, *J. Appl. Crystallogr.*, 2018, **51**, 1151–1161.
